# Strong Antiproliferative Activity Observed in Hammett-Guided Electronic Modulation of GPx-Mimetic Pathways in Aryl Selenoureas

**DOI:** 10.3390/ijms27083574

**Published:** 2026-04-16

**Authors:** Paloma Begines, Clara I. Pérez-Lage, Adrián Puerta, José M. Padrón, Óscar López, José G. Fernández-Bolaños

**Affiliations:** 1 Departamento de Química Orgánica, Facultad de Química, Universidad de Sevilla, Apartado 1203, E-41071 Seville, Spain; pbegines@us.es (P.B.); clarypl@hotmail.com (C.I.P.-L.); 2 BioLab, Instituto Universitario de Bio-Orgánica “Antonio González” (IUBO-AG), Universidad de La Laguna, c/Astrofísico Francisco Sánchez 2, E-38206 La Laguna, Spain; apuertaa@ull.es (A.P.); jmpadron@ull.es (J.M.P.)

**Keywords:** selenoureas, antioxidants, GPx-mimic, Hammett parameters, ^77^Se-NMR, antiproliferative properties

## Abstract

Organoselenium chemistry has undergone remarkable development over the past five decades, evolving from its initial association with high toxicity into a field with pivotal contributions to materials science, organic synthesis, catalysis, and Medicinal Chemistry. Among the diverse biological activities displayed by organoselenium compounds, their redox behaviour is particularly compelling, as many of these molecules act as efficient mimetics of the antioxidant enzyme glutathione peroxidase (GPx). In this work, we investigated the GPx-like activity of a series of *N*,*N*′-diaryl selenoureas toward the depletion of H_2_O_2_ and cumene hydroperoxide (CumOOH) as model ROS. Their reactivity was correlated with the electronic nature of the aryl substituents using a Hammett-type analysis, revealing a strong dependence of the reaction rate on remote electronic perturbations within the aromatic ring. Combined UV and NMR studies provided mechanistic evidence supporting a catalytic cycle in which selenoureas, operating at sub-stoichiometric loadings (1 mol%) and using a thiol as a cofactor-like molecule, can be used to efficiently scavenge ROS with half-lives of only a few minutes (~10–60 min). Furthermore, these selenoureas exhibited potent antiproliferative activity across several human solid tumour cell lines. Overall, these results offer mechanistic insight into the ROS-eliminating pathways of selenoureas and highlight their potential as chemopreventive or anticancer agents.

## 1. Introduction

Selenium, discovered in 1817 by the Swedish chemist Jöns Jacob Berzelius [1], was long regarded primarily as a toxic element [2]. Its association with poisoning episodes in humans and livestock constrained scientific interest for more than a century [3]. Historical records even describe symptoms consistent with selenium intoxication during Marco Polo’s travels through Western China in the 13th century, and some authors have speculated that selenium-rich plants (seleniferous or Se-accumulating species) may have contributed to the defeat of Lieutenant Colonel Custer at the Battle of the Little Bighorn by poisoning cavalry horses [4].

This long-standing perception underwent a profound transformation in the 1970s, when selenium was identified as an essential component of key enzymes, now known as selenoproteins, in which it is incorporated as the amino acid selenocysteine [5]. This discovery marked a turning point in our understanding the biological relevance of selenium. Over 20 selenoproteins [6] have been identified, including glutathione peroxidases (GPx) [7], thioredoxin reductases (TrxR) [8], and iodothyronine deiodinases (DIO) [9], all of which are central regulators of redox homeostasis, intracellular signalling pathways, and thyroid hormone metabolism [10]. Selenium is an essential trace micronutrient [11], and its deficiency is associated with severe pathologies [12].

In parallel with these discoveries, interest in organoselenium chemistry has grown exponentially [13]. Although there are numerous sulphur-containing compounds exhibiting valuable biological properties [14], selenium displays distinctive physicochemical properties, like greater nucleophilicity, higher polarizability, and an increased propensity for oxidation [15,16] providing unique reactivity profiles. Organoselenium chemistry has expanded substantially, and the principles of Green Chemistry have been successfully incorporated to develop environmentally friendly methods for synthesizing selenium-containing compounds [17]. Mechanochemical approaches [18], solvent-free protocols [19], flow chemistry [20], ultrasound-mediated, visible-light-mediated or electrochemically mediated reactions [21] have all contributed to this progress.

These advances have enabled the preparation of a wide array of useful synthetic building blocks [22], mimetics of natural compounds, like selenosugars [13,23], catalysts [24], and functional derivatives applicable in Materials Science [25,26], and very prominently, in the design of bioactive molecules [27]. A vast arsenal of organoselenium compounds has shown potential therapeutic relevance against numerous diseases and disorders [28,29], including cancer [30,31,32,33,34,35,36], Alzheimer’s disease [37,38,39], epilepsy [40], inflammatory diseases [41], microbial infections [42,43,44,45,46], lysosomal storage disorders [47], or osteoporosis [48], among others.

Among their various attributes, perhaps the most compelling property of organoselenium compounds is their pronounced redox activity. Their capacity to scavenge Reactive Oxygen/Nitrogen Species (ROS/RNS, respectively), including free radicals, peroxides, and hydroperoxides has become a particularly active research area, as these redox processes underlie many of the observed biological effects [49].

In this manuscript, we present an in-depth analysis of the antioxidant properties of *N*,*N*′-diaryl selenoureas, focusing on their ability to function as efficient GPx mimetics and proposing a tentative mechanistic pathway for their redox activity.

## 2. Results and Discussion

### 2.1. Design of Bioactive Molecules

Among the diverse biological activities exhibited by organoselenium compounds, their ability to modulate and deplete excessive ROS levels stands out as particularly noteworthy. The controlled reduction in overproduced reactive species can profoundly influence the management of numerous disorders and diseases, while also positioning Se-containing molecules as promising chemopreventive agents [50]. Despite this potential, the mechanisms by which seleno-compounds interact with reactive species such as ROS and RNS remain only partially understood. Gaining deeper mechanistic insight into these interactions is essential for the rational design of more potent and selective bioactive molecules.

Within this chemical family, selenoureas—selenium-based isosteres of ureas and thioureas—represent a particularly intriguing class [36,51].

**Figure 1:**
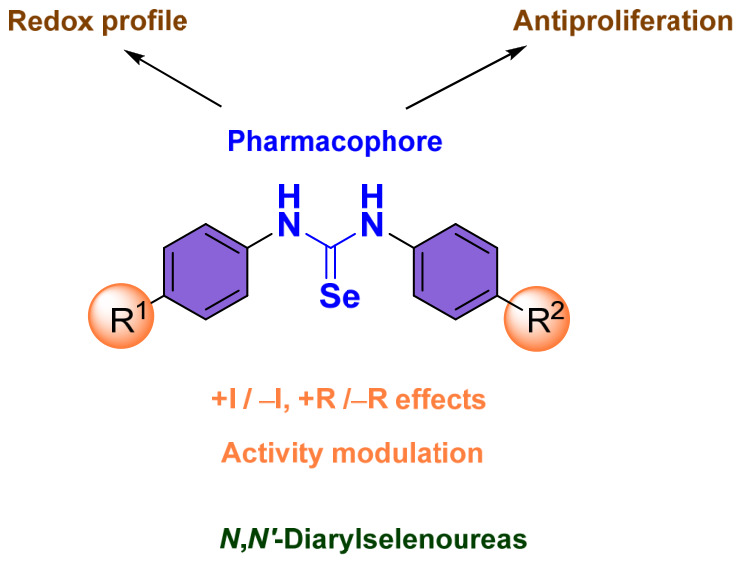
General structure of *N*,*N*′-diaryl selenoureas designed as prototypes for studying the antioxidant properties of the selenoureido motif.

Herein, we explore a simple prototype: *N*,*N*′-diaryl selenoureas (Figure 1). Our aim was to gain mechanistic insight into their ROS-eliminating behaviour, in particular, peroxides and alkyl peroxides. We hypothesized that introducing substituents with distinct stereoelectronic characteristics could fine-tune the antioxidant response of these selenoureas and provide valuable evidence regarding their underlying mode of action.

The synthetic strategy relied on coupling aryl isoselenocyanates [52] with aromatic amines. To better quantify the electronic influence of the substituents, one aromatic ring was kept unsubstituted, while the other incorporated *para*-substituents with distinct electron-donating or electron-withdrawing effects. As a more versatile approach, we initially attempted the direct coupling of aniline isoselenocyanate with various *p*-substituted anilines. However, these reactions resulted either in no detectable progress or in the formation of complex mixtures of side products.

**Scheme 1:**
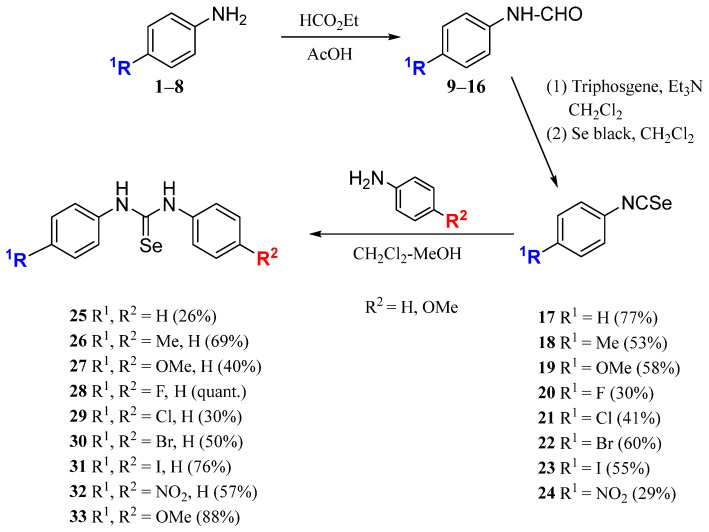
Preparation of *N*,*N*′-diaryl selenoureas **25**–**33** from commercially available anilines.

We therefore adopted an alternative and more reliable route: the individual preparation of each aniline-derived isoselenocyanate (compounds **17**–**24**, Scheme 1). These intermediates were generated through *N*-formylation of anilines **1**–**8** using ethyl formate, followed by triphosgene-mediated dehydration to the corresponding transient isocyanates, which were subsequently reacted with elemental selenium black to afford the isoselenocyanates (Scheme 1). Final coupling with aniline afforded selenoureas **25**–**32**. Using the same protocol, the symmetrical *p*-dimethoxy-substituted selenourea **33** was also successfully obtained. *N*,*N*′-Difenil selenourea **25** was previously used [53] by some of us for the selective detection of anions through the formation of mono- and bi-coordinated adducts, acting as molecular logic gates.

The synthesis of **25**–**33** typically proceeded to completion within minutes, and the products could be isolated in high purity simply by filtration from the reaction medium. Attempts to purify the materials by column chromatography led to significant decomposition, confirming that direct isolation was the most suitable workup procedure.

^1^H-NMR spectroscopy revealed that the NH protons appear markedly deshielded (δ = 9–11 ppm), consistent with the strong local anisotropic effects generated by the flanking aromatic rings and the electron-withdrawing nature of the selone group, which induces partial positive charge on the nitrogen atoms. In addition, selenoureas exhibit an enhanced hydrogen-bond-donor capacity compared to ureas and thioureas, enabling strong interactions with the solvent (DMSO-d_6_), which further contributes to the observed downfield shifts.

In the ^13^C-NMR spectra, the selone carbon resonates within a narrow range of 178–181 ppm, in line with the highly polarized C=Se bond. ^77^Se is the only NMR active isotope in NMR (7.63% isotopic abundance), endowed with a much higher sensitivity towards structural changes than ^1^H and ^13^C [54]. Indeed, ^77^Se-NMR spectroscopy proved to be substantially more sensitive to subtle structural variations, even distinguishing between conformers more efficiently than ^1^H and ^13^C spectroscopy, or reaction intermediates [55]. ^77^Se spectroscopy exhibits a large chemical shift range [56] (~3000 ppm), so it has become a highly valuable tool in structural studies. Exceptionally high chemical shifts have been recorded for sterically hindered selenoaldehydes and ketones (~2400 ppm), whereas a highly negative chemical shift was found for O=C=Se (−447 ppm) [55]. In selenoureas, extensive n→π* back-donation from the nitrogen atoms to the C=Se bond significantly stabilizes the partial double-bond character, resulting in unusually low ^77^Se chemical shifts (150–245 ppm) [55]. These reported values are in agreement with the chemical shifts found for selenoureas **25**–**33**, which exhibited resonances spanning 260–331 ppm (Table 1).

Notably, the ^77^Se chemical shifts exhibit a clear and systematic response to the electron-donating or electron-withdrawing nature of the *para*-substituents, despite the selenium atom being separated by a seven-bond distance. This remarkable long-range sensitivity underscores the exceptional efficiency of electronic transmission across the selenoureido framework.

Correlations between Hammett constants and spectroscopic parameters—particularly ^1^H and ^13^C chemical shifts—have been documented in the literature [57]. When such correlations arise, they indicate that substituent-induced variations in chemical shift are electronic in origin and therefore dependent on the donor/acceptor character of the substituents. Within the framework of linear free-energy relationships, these effects can be described by Equation (1):

δ = ρσ + δ_0_ (1)

**Equation (1).** Correlation between chemical shifts (δ) and Hammett constants (σ).

Here, ρ represents the sensitivity of the nucleus to electronic perturbations, and δ_0_ corresponds to the chemical shift of the unsubstituted aryl fragment.

**Figure 2:**
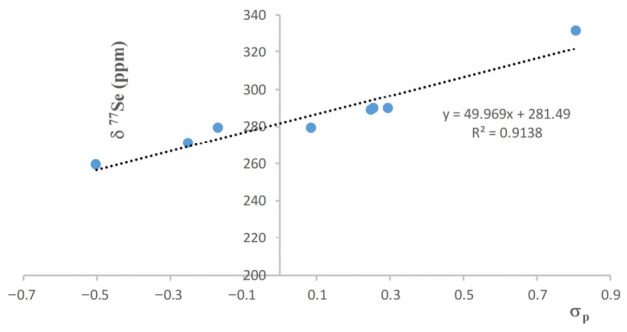
Plot correlating ^77^Se chemical shifts of selenoureas **25**–**33** with Hammett constants.

Figure 2 displays the plot of ^77^Se chemical shifts vs. the Hammett constants of *para*-substituents for selenoureas **25**–**33**. The overall Hammett constants for the *p*-substitution were used, expressed as a linear combination of inductive and resonance effects (σ*_p_* = σ_I_ + σ_R_) [58]. An excellent linear correlation was obtained. Although ^77^Se has been used in this kind of analysis for *O*/*Se*-aryl selenocarbamates [59], our results constitute the first report of a Hammett-type correlation involving ^77^Se chemical shifts in *N*,*N*-diaryl selenoureas.

### 2.2. Investigation of the Mechanistic Aspects of GPx-like Properties

Hydrogen peroxide is a strong non-radical oxidant generated in cells through both enzymatic and non-enzymatic pathways. Non-enzymatic production primarily arises from electron leakage during mitochondrial aerobic respiration, whereas the enzymatic route is dominated by the action of superoxide dismutases (SOD1, SOD2, and SOD3), which rapidly convert superoxide (O_2_•^−^) into H_2_O_2_. In parallel, alkyl hydroperoxides (ROOH) are formed predominantly through the oxidative degradation of membrane phospholipids during lipid peroxidation.

To maintain redox homeostasis, cells rely on an intricate antioxidant network, within which glutathione peroxidases (GPx) constitute a central defensive element. GPx enzymes protect against the deleterious effects of H_2_O_2_ and alkyl hydroperoxides by catalyzing their reduction to water and the corresponding alcohols, respectively. This process proceeds through a highly efficient catalytic cycle enabled by the presence of a selenocysteine residue at the active site [60]. During catalysis, the selenol group is oxidised to the selenenic acid intermediate (GPx–SeOH). Subsequent reactions with reduced glutathione (GSH), which acts as a sacrificial cofactor, regenerate the active selenol form, thereby restoring the catalytic activity of the enzyme [60].

Several families of organoselenium compounds, like selenides and selenoxides have been reported to exhibit GPx-like activity [61], enabling them to scavenge peroxide-type reactive oxygen species. Among them, ebselen (2-phenyl-1,2-benzisoselenazol-3(2*H*)-one) and its analogues constitute the most extensively studied class [62]. Even, some organotellurium exhibited GPx-mimicry properties [63]. However, in *N*,*N*′-diaryl selenoureas, the precise mechanistic pathways operating, and the influence of their structural features on GPx-like behaviour, remain poorly understood.

GPx mimicry by selenoureas **25**–**33** was evaluated using two complementary methodologies. In the first approach, the selenoureas were employed in catalytic amounts (1 mol% relative to the cofactor) in the presence of an excess of the ROS and PhSH as the cofactor, following the procedure originally described by Mugesh and co-workers [64]. The reaction progress was monitored by UV spectroscopy by tracing the increase in absorbance at 305 nm, corresponding to the formation of the oxidised form of PhSH, (PhS)_2_. As ROS models, we selected H_2_O_2_, the most relevant intracellular pro-oxidant species [65], and CumOOH, a representative mimic of natural alkyl hydroperoxides [66]. Table 2 and Table 3 summarize the reaction rates obtained from UV monitoring for selenoureas **25**–**33**, alongside the uncatalysed background reaction, as well as the corresponding half-reaction times (t_1/2_), using pro-oxidant concentrations of 10.4 mM (Table 2) and 41.6 mM (Table 3).

The data clearly demonstrate that *N*,*N*′-diaryl selenoureas **25**–**33** act as highly efficient GPx mimetics, producing substantial rate enhancements relative to the uncatalysed process. A pronounced dependence on the electronic nature of the *para*-substituents was also observed. The most active peroxide and hydroperoxide scavengers under both sets of conditions were selenoureas **27** and **33**, bearing one or two OMe groups, respectively, which provided up to 32- and 34-fold rate accelerations (334.8 and 354 µM min^−1^ vs. 10.5 µM min^−1^, Table 3).

Analysis of the data enabled the establishment of clear structure–activity relationships. Selenoureas containing electron-donating substituents at the *para* position, such as derivatives **26** (Me, moderate +I effect), **27** (OMe, strong +R effect), and especially **33** (two OMe groups), displayed substantially enhanced reaction rates. Conversely, compounds bearing electron-withdrawing groups (**28**–**32**) showed markedly lower activity, particularly nitro-substituted **32**, which exhibits strong –I and –R effects. In all cases, the selenoureas were more effective at scavenging H_2_O_2_ than cumene hydroperoxide, although substituent-dependent trends were preserved.

These results suggest that the rate-determining step of ROS reduction by selenoureas involves the development of partial positive charge. This hypothesis was further examined through a correlation analysis between the reaction rates of selenoureas **25**–**33** and the electronic properties of their *para*-substituents, quantified by Hammett σ_p_ parameters.

**Figure 3:**
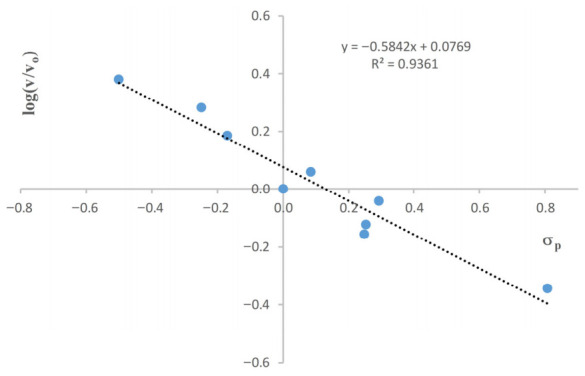
Hammett plot for the GPx-like activity of selenoureas **25**–**33** in the scavenging of H_2_O_2_. [ROS] = 10.4 mM; [Cat] = 1 mol%.

Compound **25**, lacking substituents, was used as the reference. Such physicochemical correlations provide valuable insights into reaction mechanisms. For this purpose, log(v/v_0_) was plotted against σ_p_; Figure 3 depicts such plot for [H_2_O_2_] = 10.4 mM, as a representative example. A negative slope was obtained (ρ = −0.5842), consistent with accumulation of positive charge in the rate-determining step. The magnitude of the slope indicates that the charge is unlikely to be located on the vicinal positions or on the aromatic rings, where more pronounced slopes would be expected, but rather on the selenium atom itself. Electron-donating groups, whether through +I or +R effects, stabilize this developing positive charge, thus increasing the reaction rate. Furthermore, the strong correlations observed confirm the high sensitivity of the selenium centre to electronic perturbations transmitted from a relatively distant position on the molecule. Similar results were observed for the other three conditions obtained from Table 2 and Table 3.

We further examined the mechanism underlying the GPx-like of *N*,*N*′-diaryl selenoureas activity using NMR spectroscopy. Attempts to monitor the reaction by ^77^Se-NMR were unsuccessful because of the low natural abundance of this isotope relative to ^1^H requires long acquisition times, which in turn led to catalyst decomposition under the reaction conditions. Consequently, we monitored the reaction progress by ^1^H-NMR spectroscopy, by applying slight modifications to the original methodology developed by Iwaoka and Kumakura [67]. In this approach, sub-stoichiometric amounts of the catalyst were treated with an excess of H_2_O_2_ and dithiothreitol (DTT^red^), used as a cofactor surrogate. The conversion of DTT^red^ into its oxidised form (DTT^ox^) was followed by the disappearance of the signals at δ = 2.63 and 3.67 ppm (DTT^red^) and by the emergence of new signals at δ = 2.87, 3.03, and 3.49 ppm (DTT^ox^).

**Figure 4:**
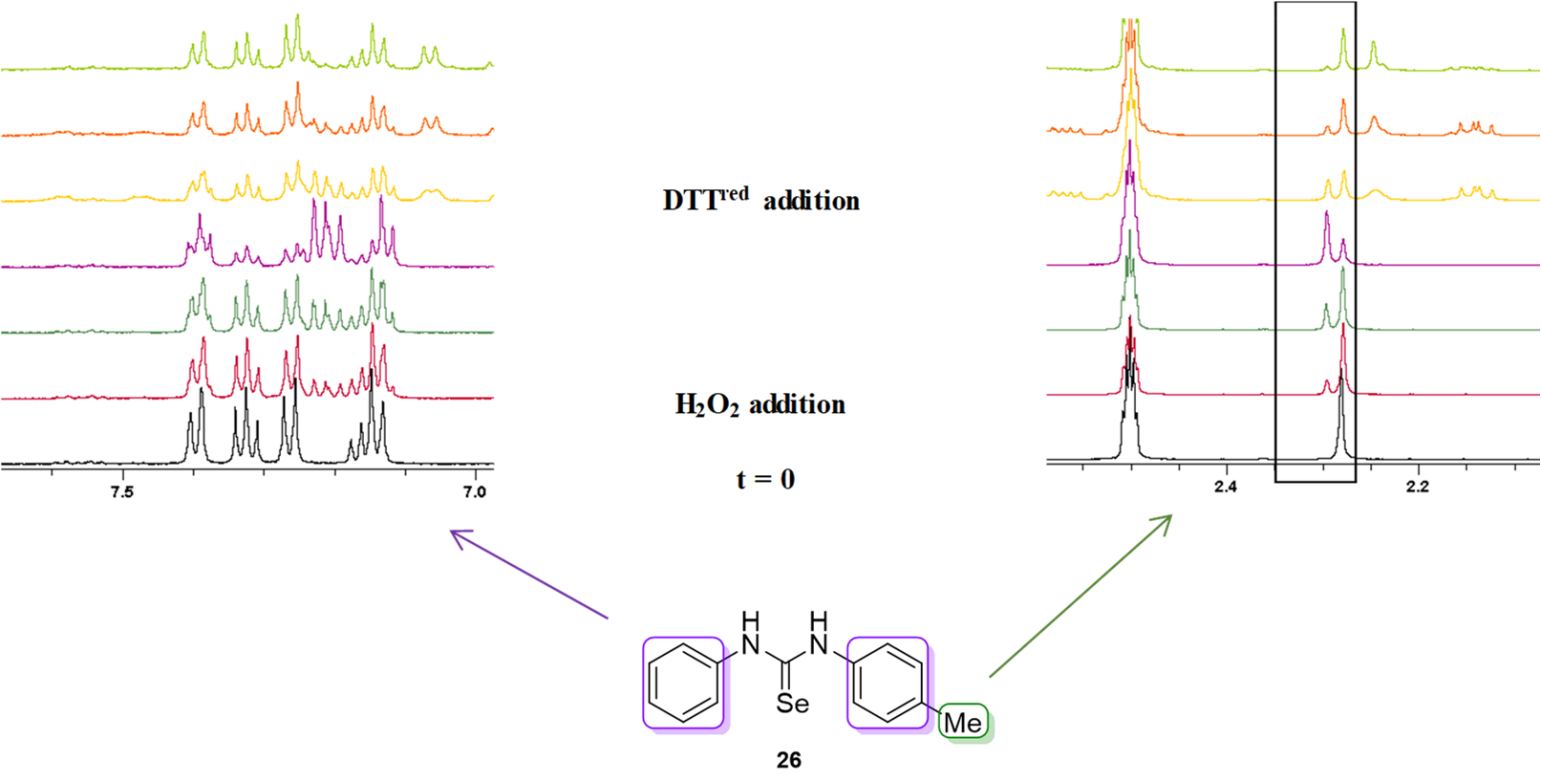
^1^H-NMR monitoring of the GPx-like activity of selenourea **26**.

Figure 4 shows the evolution of the reaction using selenourea **26** (*p*-tolyl, 1.61 µmol) as a model catalyst. Upon addition of H_2_O_2_ (176 µmol) to the selenourea solution in CD_3_OD (0.6 mL), a decrease in the intensity of the original Me signal and its gradual shift to a slightly deshielded position were observed, accompanied by the appearance of new signals in the aromatic region. Remarkably, after subsequent addition of DTT^red^ (161 µmol) to the reaction mixture, the original selenourea signals were restored, consistent with a catalytic cycle involving the selenium atom.

**Scheme 2:**
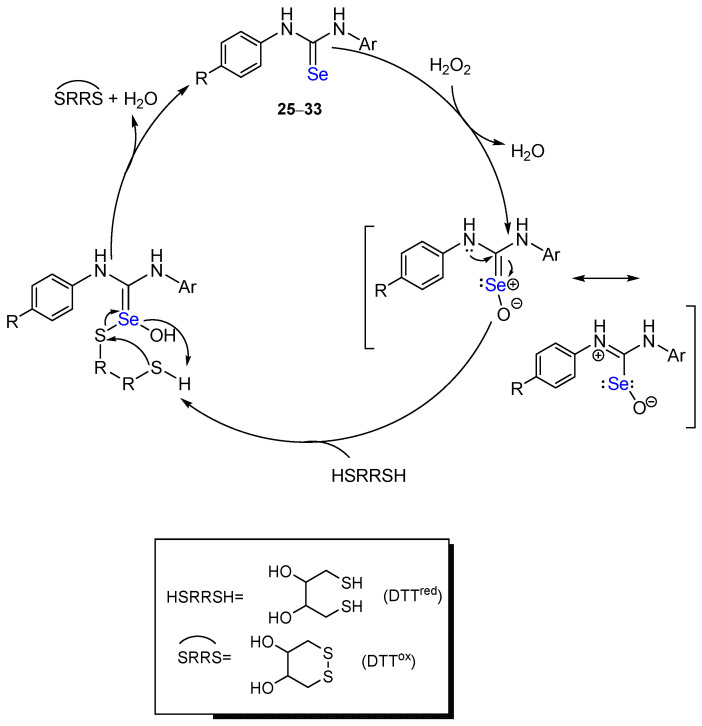
Proposed mechanism for the activity of selenoureas **25**–**33** as GPx mimetics, using DTT^red^ as a cofactor.

With all these data in hand, we propose the catalytic cycle depicted in Scheme 2 to account for the GPx-like activity of *N*,*N*′-diaryl selenoureas. The first step, identified as the rate-determining step of the process, involves the oxidation of the selenoureido moiety to generate a selenoxide-type intermediate. This intermediate bears a delocalized partial positive charge distributed between the selenium and nitrogen atoms. Such electronic distribution explains why electron-donating substituents on the aryl ring, through either inductive or resonance effects, enhance the reaction rate by stabilizing the transient selenoxide intermediate. Although we could not detect the presence of the selenoxide intermediate by spectroscopic methods, this hypothesis is in agreement with reported two-electron oxidation of selenoureas [68]. In the absence of a thiol cofactor, the transient selenoxide was reported to evolve to selenium nanoparticles.

Subsequently, this intermediate reacts with the thiol cofactor (PhSH in the UV assay or DTT^red^ in the NMR experiments) to afford a selenyl–sulfide adduct. Incorporation of a second equivalent of thiol then regenerates the parent selenourea, thereby closing the catalytic cycle. As a consequence, sub-stoichiometric amounts of the catalyst are sufficient to efficiently deplete ROS, reaching t_1/2_ values of only a few minutes.

### 2.3. Antiproliferative Properties

Selenoureas **25**–**33** were evaluated in vitro for their antiproliferative properties against a panel of six human tumour cell lines: A549 (non-small cell lung), HBL-100 (breast), HeLa (cervix), SW1573 (non-small cell lung), and the multidrug resistant cell lines T-47D (breast) and WiDr (colon). 5-Fluorouracil (5-FU) and cisplatin (CDDP) were included as reference drugs. All experiments were conducted following our implementation of the screening protocol of the US National Cancer Institute (NCI) [69], and results are summarized in Table 4.

The antiproliferative evaluation of selenoureas **25**–**33** revealed low-micromolar activities across several human cancer cell lines, positioning these compounds as potent examples of small-molecule organoselenium antiproliferative agents. Comparable activity ranges have been described for selenoureas derived from natural compounds [36], and other selenium-based chemotypes such as selenocyanate conjugates [31], or ebselen analogues [70], yet many of these require structural complexity or multitarget mechanisms to achieve low-micromolar potencies. In contrast, the *N*,*N′-*diaryl selenoureas examined here display similar or superior GI_50_ values while maintaining a structurally minimal scaffold, highlighting their favourable activity-to-complexity ratio.

The SAR trends observed for this series also resonate with prior studies in organoselenium Medicinal Chemistry, where halogenated derivatives often enhance lipophilicity and metabolic stability [71,72]. Notably, compounds **29** (*p*-Cl) and **31** (*p*-I) exhibited consistently high potency across nearly all tested lines, including multidrug-resistant models. On the contrary, the nitro derivative **32** exhibited impaired antiproliferative activity.

Conversely, it is important to highlight that the most potent GPx-mimetic selenoureas (**27** and **33**) do not necessarily translate into the most potent antiproliferative agents in all cancer cell lines, underscoring the multifaceted and context-dependent relationship between redox modulation and cancer cell viability. This divergence aligns with the growing recognition that selenium compounds may exert either protective or cytotoxic effects depending on the antioxidant nature, type of tumour, dosage, or genetics.

There is also high heterogeneity on how the tumour is affected: redox homeostasis, metabolism, immune response, or epigenetics [73]. There are examples in which antioxidants exhibit anticancer properties but there are also reports for depleting the high levels of glutathione, which in combination with photodynamic therapy might constitute another alternative in anticancer research [74].

Overall, the antiproliferative findings presented in this work expand the chemical space of bioactive organoselenium compounds and identify diaryl selenoureas as promising leads for further development. Their combination of tunable redox properties, robust GPx-like catalysis, and significant antiproliferative activity suggests that these derivatives may serve as multifunctional agents capable of modulating oxidative stress or triggering ROS-related cell death pathways. Future studies will focus on elucidating their intracellular mode of action, evaluating their selectivity to determine their translational potential.

## 3. Materials and Methods

### 3.1. Chemistry

#### 3.1.1. General Methods

TLC (thin-layer chromatography) was performed using aluminium-coated sheets (Merck 60 F_254_, Darmstadt, Germany), 0.25 mm width. Each eluent is indicated in the experimental procedures. Spots were visualized by UV light (λ = 254 nm) and by charring with 10% ethanolic vanillin containing 1% H_2_SO_4_ or with 5% ethanolic phosphomolybdic acid. Column chromatography purifications were performed using silica gel stationary phase (Merck 60, particle size 40–63 μm), eluting by gravity or with mild pressure. Eluents are indicated in each case. NMR spectra were acquired in the Centro de Investigación, Tecnología e Innovación de la Universidad de Sevilla (CITIUS), using Bruker Avance III 300 and 500 spectrometers (300.1 MHz and 500 MHz for ^1^H; 75.5 and 125.7 MHz, respectively, for ^13^C) (Billerica, Massachusetts, USA), and DMSO-*d*_6_ as solvent (VWR Chemicals, Radnor, PA, USA). Chemical shifts (δ) are expressed in ppm, and coupling constants (*J*) are expressed in Hz. Residual signals from the solvent were used as internal references [75] for the calibration (2.50 ppm for ^1^H and 39.5 ppm for ^13^C). ^1^H, ^13^C and ^77^Se NMR spectra are provided in the Supporting Information file (Figures S1–S24). Mass spectra were registered using a Qexactive spectrometer using Electrospray Ionization (ESI). Sample injection (MeOH as solvent) was performed using UHPLC without column (50 μL and acquisition for 3 min at 0.200 mL/min). Acquisition was carried out with full scan at 60,000 resolution. Capillary temperature was 350 °C and the source voltage, 3.5 kV. Spectra were registered under positive mode and calibrated using the Pierce™ LTQ Velos ESI Positive Ion Calibration Solution (ThermoFisher Scientific, Waltham, MA, USA).

#### 3.1.2. General Procedure for the Preparation of Selenoureas **25**–**33**

To a solution of the corresponding isoselenocyanate (0.28 mmol) in a 1.5:1 CH_2_Cl_2_–MeOH mixture (5 mL), aniline (26 µL, 0.28 mmol, 1.0 equiv.) or *p*-anisidine (32.8 µL, 0.28 mmol, 1.0 equiv., for **33**), was added and stirred at room temperature for 5 min. The reaction was then stirred at 40 °C for other 5 min. The reaction medium was then cooled, filtered and washed with cyclohexane, or separated by centrifugation.

*N*-(*p*-methylphenyl)-*N*′*-*phenyl-selenourea (**26**). *p*-Tolyl isoselenocyanate (**18**) (55.0 mg, 0.28 mmol) was used. The precipitate was filtered and washed with cyclohexane, yielding **26** as a reddish solid. Yield: 55.9 mg (69%); m.p.: 175 °C; *R*_F_ 0.59 (20:1 cyclohexane–EtOAc); ^1^H-NMR (500 MHz, DMSO-*d*_6_) δ: 10.05 (s, 1H, NH), 10.02 (s, 1H, NH), 7.39 (m, 2H, H-3′, H-5′), 7.33 (m, 2H, H-3, H-5), 7.26 (m, 2H, H-2′, H-6′), 7.15 (m, 3H, H-2, H-4, H-6), 2.28 (s, 3H, CH3) ppm; ^13^C-NMR (125.7 MHz, DMSO-*d*_6_) δ: 178.5 (CSe), 139.7 (C-1), 137.1 (C-1′), 134.5 (C-4′), 129.0 (C-3′, C5′), 128.5 (C-3, C-5), 125.1 (C-4), 124.8 (C-2, C-6), 124.7 (C-2′, C-6′), 20.6 (CH3) ppm; ^77^Se-NMR (95 MHz, DMSO-*d*_6_) δ: 279.4 ppm; HRESIMS *m*/*z* calcd. for C_14_H_15_N_2_^80^Se [M+H]^+^: 291.0395, found 291.0397.

*N*-(*p*-Methoxyphenyl)-*N*′-phenyl-selenourea (**27**). *p*-Methoxyphenyl isoselenocyanate (**19**) (59.4 mg, 0.28 mmol) was used. The precipitate was filtered and washed with cyclohexane, yielding **27** as a white solid. Yield: 34.2 mg (40%); m.p.: 160 °C; *R*_F_ 0.39 (20:1 cyclohexane–EtOAc); ^1^H-NMR (500 MHz, DMSO-*d*_6_) δ: 9.95 (m, 2H, 2 NH), 7.41 (m, 2H, Ar-H), 7.35 (m, 2H, Ar-H), 7.28 (m, 2H, Ar-H), 7.16 (m, 1H, Ar-H), 6.9 (m, 2H, Ar-H), 3.75 (s, 3H, OCH_3_) ppm; ^13^C-NMR (125.7 MHz, DMSO-*d*_6_) δ: 178.6 (CSe), 157.0 (C-4′), 139.7, 132.4 (C-1, C-1′), 128.5 (x2) (Ar-C), 126.8 (x2) (Ar-C), 125.1 (Ar-C), 124.8 (x2) (Ar-C), 113.8 (x2) (Ar-C), 55.3 (OCH_3_) ppm; ^77^Se-NMR (95 MHz, DMSO-*d*_6_) δ: 271.7 ppm; HRESIMS *m*/*z* calcd. for C_14_H_15_N_2_O^80^Se [M+H]^+^: 307.0344, found 307.0343.

*N*-(*p*-Fluorophenyl)-*N*′-phenyl-selenourea (**28**). *p*-Fluorophenyl isoselenocyanate (**20**) (56.0 mg, 0.28 mmol) was used. The precipitate was filtered and washed with cyclohexane, yielding **28** as a pinkish-white solid. Yield: 82.1 mg (quant.); m.p.: 145 °C; *R*_F_ 0.61 (20:1 cyclohexane–EtOAc). ^1^H-NMR (500 MHz, DMSO-*d*_6_) δ: 10.14 (s, 1H, NH), 10.06 (s, 1H, NH), 7.39 (m, 4H, Ar-H), 7.34 (m, 2H, Ar-H), 7.17 (m, 3H, Ar-H) ppm; ^13^C-NMR (125.7 MHz, DMSO-*d*_6_) δ: 179.5 (CSe), 160.1 (d, ^1^*J*_C,F_ = 244 Hz, C-4′,), 140.0 (C-1), 136.6 (brd, ^4^*J*_C,F_ = 1.7 Hz, C-1′), 129.1 (x2) (Ar-C), 127.8 (x2) (Ar-C), 127.8 (d, ^3^*J*_C,F_ = 8.4 Hz, C-6′/C-2′), 125.8 (Ar-C), 125.3 (x2) (Ar-C), 115.6 (^2^*J*_C,F_ = 22.6 Hz, C-5′/C-3′) ppm; ^77^Se-NMR (95 MHz, DMSO-*d*_6_) δ: 279.5 ppm; HRESIMS *m*/*z* calcd. for C_13_H_12_FN_2_^80^Se [M+H]^+^: 295.0144, found 295.0145.

*N*-(*p*-Chlorophenyl)-*N*′-phenylselenourea (**29**). *p*-Chlorophenyl isoselenocyanate (**21**) (60.6 mg, 0.28 mmol) was used. The precipitate was filtered and washed with cyclohexane, yielding **29** as a pinkish-white solid. Yield: 26.0 mg (30%); m.p.: 170 °C; *R*_F_ 0.60 (20:1 cyclohexane–EtOAc). ^1^H-NMR (500 MHz, DMSO-*d*_6_) δ: 10.19 (s, 1H, NH), 10.10 (s, 1H, NH), 7.45–7.32 (m, 8H, Ar-H), 7.18 (m, 1H, Ar-H) ppm; ^13^C-NMR (125.7 MHz, DMSO-*d*_6_) δ: 178.8 (CSe), 139.4, 138.7 (C-1, C-1′), 129.1 (Ar-C), 128.5 (x2) (Ar-C), 128.2 (x2) (Ar-C), 126.4 (x2) (Ar-C), 125.2 (Ar-C), 124.5 (x2) (Ar-C)ppm; ^77^Se-NMR (95 MHz, DMSO-*d*_6_) δ: 288.9 ppm; HRESIMS *m*/*z* calcd. for C_13_H_12_ClN_2_^80^Se [M+H]^+^: 310.9849, found 310.9847.

*N*-Bromophenyl-*N*′-phenylselenourea (**30**). *p*-Bromophenyl isoselenocyanate (**22**) (73.1 mg, 0.28 mmol) was used. The precipitate was filtered and washed with cyclohexane, yielding **30** as a white solid. Yield: 32.9 mg (50%); m.p.: 175 °C; *R*_F_ 0.62 (20:1 cyclohexane–EtOAc); ^1^H-NMR (500 MHz, DMSO-*d*_6_) δ: 10.25 (s, 1H, NH), 10.14 (s, 1H, NH), 7.51 (m, 2H, Ar-H), 7.36 (m, 6H, Ar-H), 7.18 (m, 1H, Ar-H) ppm; ^13^C-NMR (125.7 MHz, DMSO-*d*_6_) δ: 178.8 (CSe), 139.5, 139.2 (C-1, C-1′), 131.3 (x2) (Ar-C), 128.6 (x2) (Ar-C), 126.8 (x2) (Ar-C), 125.3 (Ar-C), 124.6 (x2) (Ar-C), 117.4 (Ar-C) ppm; ^77^Se-NMR (95 MHz, DMSO-*d*_6_) δ: 290.1 ppm; HRESIMS *m*/*z* calcd. for C_13_H_11_^79^BrN_2_Na^80^Se [M+Na]^+^: 376.9163, found 376.9154; HRESIMS *m*/*z* calcd. for C_13_H_11_^81^BrN_2_Na^80^Se [M+Na]^+^: 378.9143, found 378.9140.

*N*-(*p*-Iodophenyl)-*N*′-phenylselenourea (**31**). *p*-Iodophenyl isoselenocyanate (**23**) (86.2 mg, 0.28 mmol) was used. The precipitate was filtered and washed with cyclohexane, yielding **31** as a pinkish-white solid. Yield: 85.4 mg (76%); m.p.: 177 °C; *R*_F_ 0.70 (20:1 cyclohexane–EtOAc); ^1^H-NMR (500 MHz, DMSO-*d*_6_) δ: 10.25 (s, 1H, NH), 10.15 (s, 1H, NH), 7.67 (m, 2H, Ar-H), 7.36 (m, 4H, Ar-H), 7.20 (m, 3H, Ar-H) ppm; ^13^C-NMR (125.7 MHz, DMSO-*d*_6_) δ: 178.7 (CSe), 139.7, 139.5 (C-1, C-1′), 137.2 (x2) (Ar-C), 128.6 (x2) (Ar-C), 126.9 (x2) (Ar-C), 125.3 (Ar-C), 124.6 (x2) (Ar-C), 89.6 (C-4) ppm; ^77^Se-NMR (95 MHz, DMSO-*d*_6_) δ: 290.1 ppm; HRESIMS *m*/*z* calcd. for C_13_H_12_IN_2_^80^Se [M+H]^+^: 402.9205, found 402.9199.

*N*-(*p*-Nitrophenyl)-*N*′-phenyl-selenourea (**32**). *p*-Nitrophenyl isoselenocyanate (**24**) (63.6 mg, 0.28 mmol) was used. The precipitate was separated by centrifugation, yielding **32** as a yellow solid. Yield: 51.1 mg (57%); m.p.: 157 °C; *R*_F_ 0.21 (20:1 cyclohexane–EtOAc); ^1^H-NMR (500 MHz, DMSO-*d*_6_) δ: 10.71 (s, 1H, NH), 10.60 (s, 1H, NH), 8.19 (m, 2H, Ar-H), 7.75 (m, 2H, Ar-H), 7.42 (m, 2H, Ar-H), 7.37 (m, 2H, Ar-H), 7.21 (m, 1H, Ar-H) ppm; ^13^C-NMR (125.7 MHz, DMSO-*d*_6_) δ: 179.3 (CSe), 146.3 (Ar-C), 142.9 (Ar-C), 139.3 (Ar-C), 128.7 (x2) (Ar-C), 125.6 (x2) (Ar-C), 124.4 (x2) (Ar-C), 124.2 (x2) (Ar-C), 123.0 (x2) (Ar-C) ppm; ^77^Se-NMR (95 MHz, DMSO-*d*_6_) δ: 331.5 ppm; HRESIMS *m*/*z* calcd. for C_13_H_12_N_3_O_2_^80^Se [M+H]^+^: 322.0089, found 322.0087.

*N*,*N*′-Bis(*p*-methoxyphenyl)selenourea (**33**). *p*-Methoxyphenyl isoselenocyanate (**19**) (59.4 mg, 0.28 mmol) and *p*-anisidine (32.8 µL, 0.28 mmol, 1.0 equiv.) were used. The precipitate was filtered and washed with cyclohexane, yielding **33** as a light violet solid. Yield: 82.8 mg (88%); m.p.: 186–189 °C; *R*_F_ 0.22 (20:1 cyclohexane–EtOAc); ^1^H-NMR (500 MHz, DMSO-*d*_6_) δ: 9.75 (s, 2H, 2NH), 7.24 (m, 4H, H-3, H-5, H-3′, H-5′), 6.89 (m, 8H, H-2, H-6, H-2′, H-6′), 3.74 (s, 6H, 2OCH_3_) ppm; ^13^C-NMR (125.7 MHz, DMSO-*d*_6_) δ: 178.7 (CSe), 157.0 (C-4, C-4′), 132.5 (C-1, C-1′), 126.9 (x4) (Ar-C), 113.7 (x4) (Ar-C), 55.2 (2OCH_3_) ppm; ^77^Se-NMR (95 MHz, DMSO-*d*_6_) δ: 259.7 ppm; HRESIMS *m*/*z* calcd. for C_15_H_17_N_2_O_2_^80^Se [M+H]^+^: 337.0450, found 337.0447; HRESIMS *m*/*z* calcd. for C_15_H_16_N_2_NaO_2_^80^Se [M+Na]^+^: 359.0269, found 359.0264.

### 3.2. Antioxidant Assays

#### 3.2.1. GPx-like Activity by UV Spectroscopy

The procedure originally reported by Mugesh and co-workers [64] was followed. Methanolic solutions of PhSH, ROS (H_2_O_2_ or CumOOH) and selenoureas were initially fixed at 10.0, 10.4/41.6 and 0.1 mM, respectively. Increase in absorbance values was monitored at 305 nm for 180 s, in the presence and in the absence (background reaction) of the organoselenium catalyst. Abs. (v) vs. t (s) was plotted and the velocity (slope of the plot) was obtained after correction with the background reaction. For (PhSe)_2_, the reported molar extinction coefficient was used (ε = 1.24 × 10^3^ M^−1^ cm^−1^).

#### 3.2.2. GPx-like Activity by NMR Spectroscopy

The procedure originally reported by Iwaoka and Kumakura [67] was followed. DTT^red^ (25 mg, 0.16 mmol) and the corresponding selenoureas (1.61 µmol, 1 mol%) were dissolved in CD_3_OD (0.6 mL). Reaction was initiated by addition of 30% H_2_O_2_ (18 µL, 0.18 mmol) and disappearance of CH protons of DTT^red^ (δ = 2.63, 3.68 ppm), and appearance of CH protons of the cyclic disulfide DTT^ox^ (δ = 2.87, 3.03, 3.51 ppm) was monitored. Velocity can be quantified by calculating the relative integral of DTT^red^/DTT^ox^ over time.

### 3.3. Antiproliferative Activity

#### 3.3.1. Cell Lines and Culture

The same conditions as previously reported were used [69]. The human cancer cell lines A549, HBL-100, and T-47D, as well as HeLa, were provided by Dr. Raimundo Freire (Hospital Universitario de Canarias, Tenerife, Canary Islands). The lung cancer cell lines SW1573 and WiDr were provided by Prof. G. J. Peters (VU University Medical Center, Amsterdam, The Netherlands). Cells were grown in RPMI-1640 medium containing 5% fetal bovine serum (FBS), 2 mM l-glutamine, 100 U/mL of penicillin G, and 0.1 mg/mL of streptomycin at 37 °C in a 95% humidified atmosphere of 5% CO_2_. Cells were maintained in culture in 60 mm cell culture dishes in growth medium (10 mL) and passaged twice weekly.

#### 3.3.2. Antiproliferative Tests

The same conditions as previously reported were used [69,76]. The antiproliferative activity of compounds was tested using our implementation of the protocol of the National Cancer Institute (NCI) of the USA. The following seeding densities (cells per well) were used: 2500 (A549, HBL-100, HeLa, and SW1573) and 5000 (T-47D and WiDr). Stock solutions of inhibitors (40 mM) were prepared in pure DMSO (400 times the maximum test concentration). For each test compound, the cells were exposed for a period of 48 h to serial decimal dilutions in cell culture medium of the test compounds (0.001–100 μM). For each product, GI_50_ values were calculated according to the NCI formulas (n = 3; data are expressed as mean ± SD).

## 4. Conclusions

This work demonstrates that *N*,*N***′**-diaryl selenoureas constitute a highly versatile class of redox-active small molecules capable of efficiently mimicking the catalytic behaviour of glutathione peroxidase (GPx) for scavenging peroxides. Through the combined use of kinetic assays, NMR monitoring, and Hammett-type analyses, we established that their reactivity towards H_2_O_2_ and cumene hydroperoxide is strongly dictated by the electronic nature of the *para*-substituents. The excellent linear free-energy correlations obtained reveal an exceptional long-range electronic communication across the selenoureido framework, despite the seven-bond separation between the substituent and the selenium centre.

The mechanistic data support a catalytic cycle initiated by the oxidation of the selenoureido moiety to a transient selenoxide-like species in which a partial positive charge develops on selenium. This step, identified as rate-determining, is significantly accelerated by electron-donating substituents that stabilise the emerging positive charge, whereas electron-withdrawing groups exert the opposite effect. The restoration of the initial selenourea upon reaction with a thiol cofactor, as observed in NMR experiments, confirms the operation of a robust Se(II)/Se(IV) redox cycle analogous to that operating in natural GPx, but with the key advantage of tunability through rational electronic modulation.

From a biological standpoint, several members of this family exhibited low-micromolar antiproliferative activity across a diverse panel of human tumour cell lines, outperforming reference anticancer drugs in some of the selected models, including multidrug-resistant lines. This dual behaviour, potent catalytic antioxidant activity and significant antiproliferative effects, highlights diaryl selenoureas as promising multifunctional scaffolds. The divergence observed between GPx-like efficiency and antiproliferative potency across the series also underscores the nuanced and context-dependent role of redox modulation in cancer biology, suggesting that fine-tuning the electronic profile of these compounds could selectively bias them towards antioxidant, pro-oxidant, or cytotoxic outcomes.

Overall, this study not only expands the mechanistic understanding of selenoureas as GPx mimics but also provides a rational framework for their further optimisation. Their straightforward synthesis, strong and predictable electronic responsiveness, and favourable biological profiles position these compounds as valuable platforms for the development of next-generation catalytic antioxidants and potential anticancer agents. Future efforts should include identification and characterization of reactive intermediates, evaluation of stability and redox behaviour under physiological conditions, and in vivo validation to fully assess their therapeutic potential.

## Figures and Tables

**Table 1:** Hammett constants and ^77^Se chemical shifts for selenoureas **25**–**33**.

Compound	Inductive Hammett Constant (σ_I_)	Resonance Hammett Constant (σ_R_)	Overall Hammett Constant (σ_p_)	δ (^13^C, ppm)	δ (^77^Se, ppm)
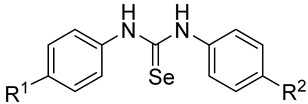					
**25** (R^1^=R^2^=H)	0.0	0.0	0.0	181.0	284.6
**26** (R^1^=Me, R^2^=H)	0.0085	−0.1781	−0.1696	178.5	279.4
**27** (R^1^=OMe, R^2^=H)	0.3962	−0.6465	−0.2503	178.6	271.7
**28** (R^1^=F, R^2^=H)	0.5305	−0.4447	0.0858	179.5	279.5
**29** (R^1^=Cl, R^2^=H)	0.4692	−0.2212	0.248	178.8	288.9
**30** (R^1^=Br, R^2^=H)	0.4963	−0.2421	0.2542	178.8	290.1
**31** (R^1^=I, R^2^=H)	0.3968	−0.1031	0.2937	178.7	290.1
**32** (R^1^=NO_2_, R^2^=H)	0.6357	0.1708	0.8065	179.3	331.5
**33** (R^1^=R^2^=OMe)	2 × 0.3962	2 × (−0.1781)	−0.5006	178.7	259.7

**Table 2:** GPx-like activity of selenoureas **25**–**33** (PhSH method) ^a^.

H_2_O_2_			CumOOH	
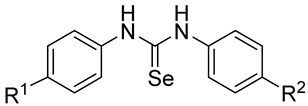				
**Compound**	**v (µM min^−1^)**	**t_1/2_ (min)**	**v (µM·min^−1^)**	**t_1/2_ (min)**
Background	13.46 ± 1.1	349.2	8.4 ± 0.9	549.0
**25** (R^1^=R^2^=H)	99.4 ± 12.4	46.8	44.2 ± 8.7	110.4
**26** (R^1^=Me, R^2^=H)	152.5 ± 8.1	25.2	74.6 ± 8.9	63.0
**27** (R^1^=OMe, R^2^=H)	190.4 ± 10.0	21.1	106.9 ± 5.4	41.4
**28** (R^1^=F, R^2^=H)	114.2 ± 7.7	41.4	43.7 ± 7.5	107.4
**29** (R^1^=Cl, R^2^=H)	69.2 ± 3.4	60.6	48.5 ± 4.5	99.0
**30** (R^1^=Br, R^2^=H)	74.8 ± 6.0	54.6	41.6 ± 1.0	113.4
**31** (R^1^=I, R^2^=H)	90.4 ± 8.6	36.6	40.0 ± 1.1	104.4
**32** (R^1^=NO_2_, R^2^=H)	44.9 ± 7.9	103.8	N.D. ^b^	N.D. ^b^
**33** (R^1^=R^2^=OMe)	239.6 ± 3.0	18.0	141.5 ± 8.2	31.8

^a^ Reaction conditions: [PhSH] = 10.0 mM, [H_2_O_2_] = [CumOOH] = 10.4 mM, [Cat] = 0.1 mM. Solvent = DMSO. ^b^ Not determined.

**Table 3:** GPx-like activity of selenoureas **25**–**33** (PhSH method) for high ROS concentrations ^a^.

H_2_O_2_			CumOOH	
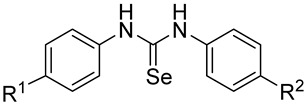				
**Compound**	**v (µM min^−1^)**	**t_1/2_ (min)**	**v (µM·min^−1^)**	**t_1/2_ (min)**
Background	10.5 ± 0.2	429.6	35.1 ± 1.2	127.8
**25** (R^1^=R^2^=H)	137.2 ± 2.9	30.6	85.6 ± 0.7	52.8
**26** (R^1^=Me, R^2^=H)	222.2 ± 4.6	18.6	123.0 ± 3.9	36.0
**27** (R^1^=OMe, R^2^=H)	334.8 ± 8.5	11.4	193.5 ± 1.3	22.8
**28** (R^1^=F, R^2^=H)	136.5 ± 1.3	30.6	125.8 ± 1.7	35.4
**29** (R^1^=Cl, R^2^=H)	72.0 ± 0.6	58.8	70.5 ± 0.5	63.6
**30** (R^1^=Br, R^2^=H)	63.1 ± 0.6	68.4	67.6 ± 1.0	67.2
**31** (R^1^=I, R^2^=H)	71.6 ± 3.1	59.4	74.5 ± 1.6	60.0
**32** (R^1^=NO_2_, R^2^=H)	N.D. ^b^	N.D. ^b^	N.D. ^b^	N.D. ^b^
**33** (R^1^=R^2^=OMe)	354.0 ± 14.7	10.2	245.0 ± 10.4	16.8

^a^ Reaction conditions: [PhSH] = 10.0 mM, [H_2_O_2_] = [CumOOH] = 41.6 mM, [Cat] = 0.1 mM. Solvent = DMSO. ^b^ Not determined.

**Table 4:** Antiproliferative properties (GI_50_, µM) for *N*,*N*′-diaryl selenoureas **25**–**33**.

Compound	A549 (Lung)	HBL-100 (Breast)	HeLa (Cervix)	SW1573 (Lung)	T-47D (Breast)	WiDr (Colon)
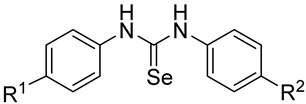						
**25** (R^1^=R^2^=H)	3.4 ± 0.3	3.1 ± 0.4	6.2 ± 0.8	4.1 ± 1.7	3.5 ± 0.4	5.1 ± 0.8
**26** (R^1^=Me, R^2^=H)	3.2 ± 0.4	3.4 ± 0.5	6 ± 1	3.2 ± 0.4	3.3 ± 0.2	4.8 ± 0.7
**27** (R^1^=OMe, R^2^=H)	3.4 ± 0.6	4.3 ± 0.7	4.2 ± 1.4	3.5 ± 0.1	3.5 ± 0.5	4.4 ± 0.4
**28** (R^1^=F, R^2^=H)	3.1 ± 0.2	3.0 ± 0.4	3.9 ± 0.3	2.1 ± 0.2	3.2 ± 0.1	4.1 ± 0.3
**29** (R^1^=Cl, R^2^=H)	2.8 ± 0.8	2.0 ± 0.5	3.0 ± 0.1	2.0 ± 0.5	3.0 ± 0.5	4.0 ± 0.2
**30** (R^1^=Br, R^2^=H)	18 ± 0.3	36 ± 3.2	27 ± 11	19 ± 3.1	27 ± 1.6	35 ± 4.2
**31** (R^1^=I, R^2^=H)	2.8 ± 0.2	2.2 ± 0.1	3.3 ± 0.1	2.3 ± 0.2	3.0 ± 0.4	4.2 ± 0.5
**32** (R^1^=NO_2_, R^2^=H)	16 ± 0.4	15 ± 5.9	8.9 ± 0.8	2.3 ± 0.4	19 ± 4.2	17 ± 1.5
**33** (R^1^=R^2^=OMe)	2.7 ± 0.3	11 ± 4.5	>100	3.2 ± 0.1	9.3 ± 3.3	4.2 ± 0.4
**5-FU**	2.2 ± 0.3	4.4 ± 0.7	16 ± 5	3.3 ± 1.2	43 ± 16	49 ± 7
**CDDP**	4.9 ± 0.2	1.9 ± 0.2	1.8 ± 0.5	2.7 ± 0.4	17 ± 3	26 ± 4

## Data Availability

The original contributions presented in this study are included in the article/Supplementary Material. Further inquiries can be directed to the corresponding authors.

## Supplementary Materials

The following supporting information can be downloaded at https://www.mdpi.com/article/10.3390/ijms27083574/s1.

## Abbreviations

The following abbreviations are used in this manuscript:

Brd	Broad doublet
calcd.	Calculated
CDDP	*cis*-Diamminedichloroplatinum(II), Cisplatin
CumOOH	Cumene hydroperoxide
D	Doublet
DIO	Iodothyronine deiodinases
DTT^ox^	Oxidised dithiothreitol
DTT^red^	Reduced dithiothreitol
equiv.	Equivalents
ESI	Electrospray ionization
GI_50_	Concentration causing 50% growth inhibition
GSH	Glutathione (reduced form)
GPx	Glutathione peroxidase
HRESIMS	High-resolution electrospray ionization mass spectrometry
M	Multiplet
m.p.	Melting point
NCI	National Cancer Institute (USA)
ROS	Reactive oxygen species
RNS	Reactive nitrogen species
S	Singlet
SOD1/2/3	Superoxide dismutases
TLC	Thin-layer chromatography
