# Supplementary material for: Strong Antiproliferative Activity Observed in Hammett-Guided Electronic Modulation of GPx-Mimetic Pathways in Aryl Selenoureas
Source: Int J Mol Sci. 2026 Apr 16;27(8):3574. doi: 10.3390/ijms27083574 (PMC13115858; doi:10.3390/ijms27083574)
Supplement: Supplementary file 1 [file ijms-27-03574-s001.zip › ijms-4222488-supplementary.pdf]

Strong Antiproliferative Activity Observed in Hammett-Guided Electronic Modulation of GPx-Mimetic Pathways in Aryl Selenoureas

Paloma Begines [1], Clara I. Pérez-Lage [1], Adrián Puerta [2], José M. Padrón [2], Óscar López [1,*] and José G. Fernández-Bolaños [1,*]

[1] Departamento de Química Orgánica, Facultad de Química, Universidad de Sevilla, Apartado 1203, E-41071 Seville, Spain; pbegines@us.es (P.B.); claryp1@hotmail.com (C.I.P.-L.)
[2] BioLab, Instituto Universitario de Bio-Orgánica "Antonio González" (IUBO-AG), Universidad de La Laguna, c/Astrofísico Francisco Sánchez 2, E-38206 La Laguna, Spain; apuertaa@ull.es (A.P.); jmpadron@ull.es (J.M.P.)
* Correspondence: osc-lopez@us.es (Ó.L.); bolanos@us.es (J.G.F.-B.)

^{1}H-, ^{13}C- and ^{77}Se-NMR spectra of compounds **26–33 (Figures S1-24)**……………………………………………………………..………………………...2–17

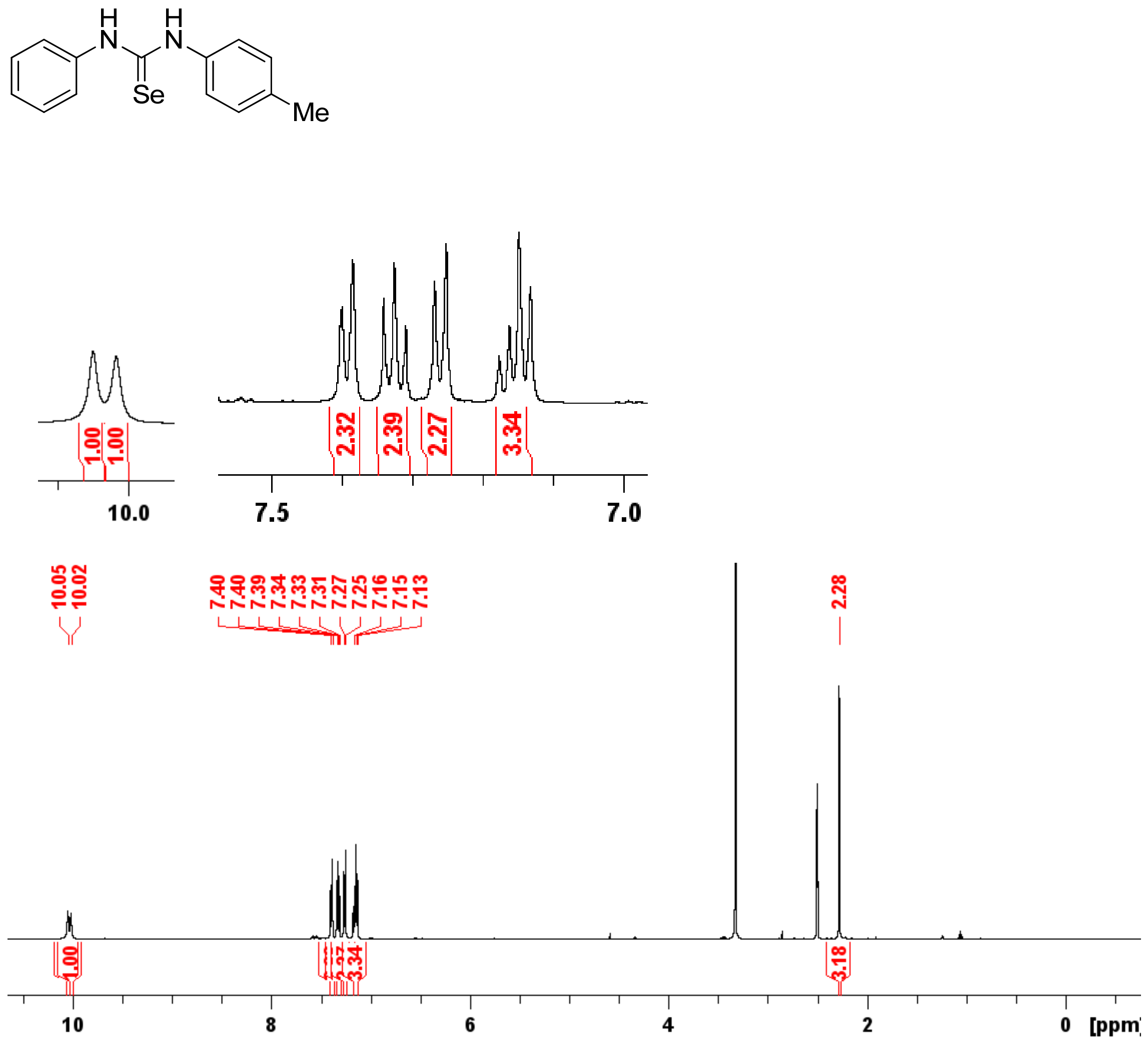

Figure S1. ^{1}H-NMR spectrum of compound **26** (500 MHz, DMSO-d_6)

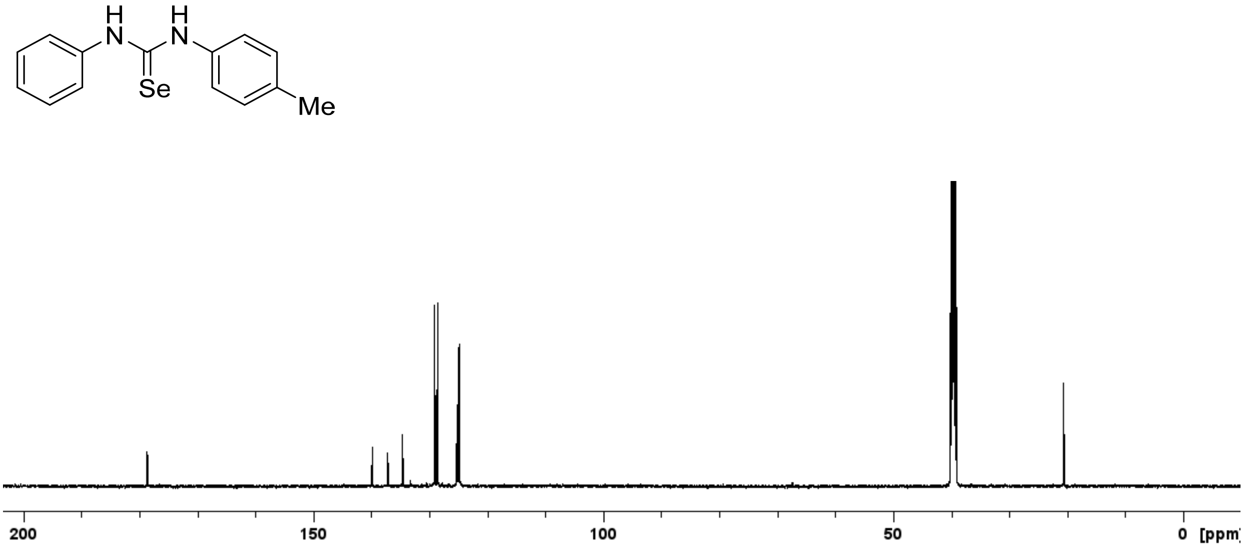

Figure S2. ^{13}C-NMR spectrum of compound **26** (125.7 MHz, DMSO-d_6)

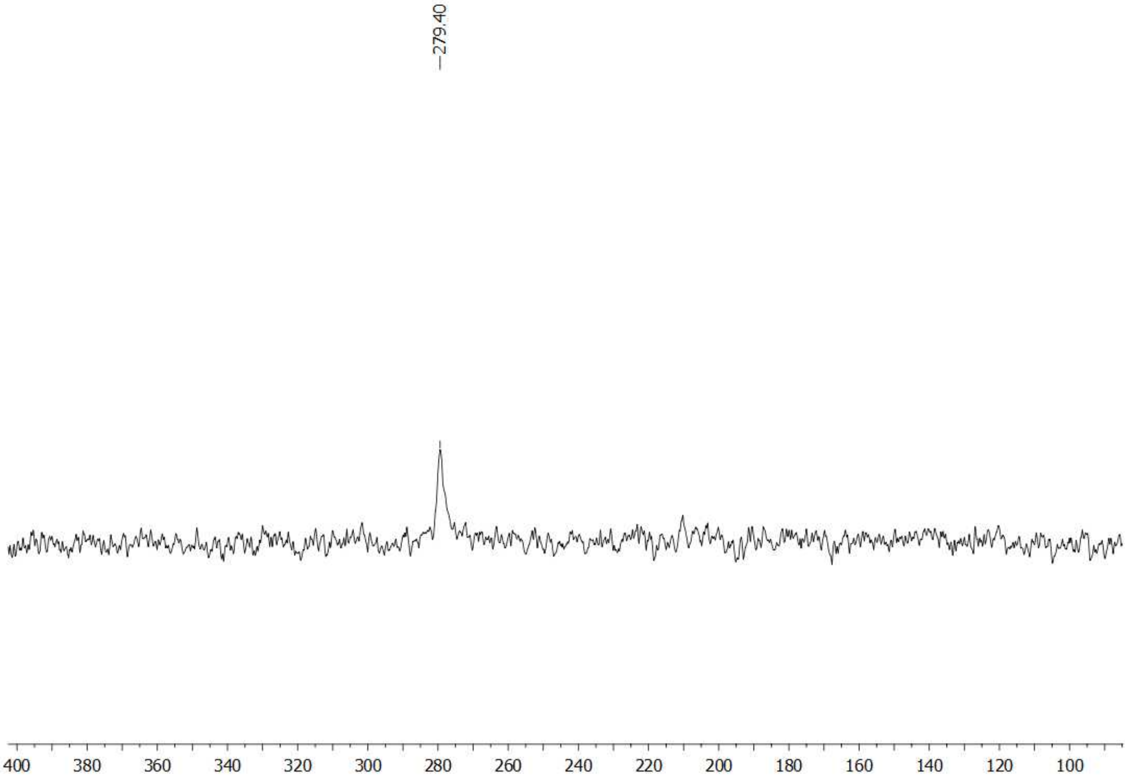

Figure S3. ^{77}Se-NMR spectrum of compound **26** (95 MHz, DMSO-d_6)

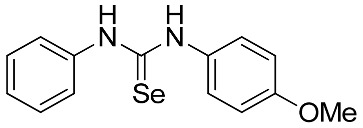

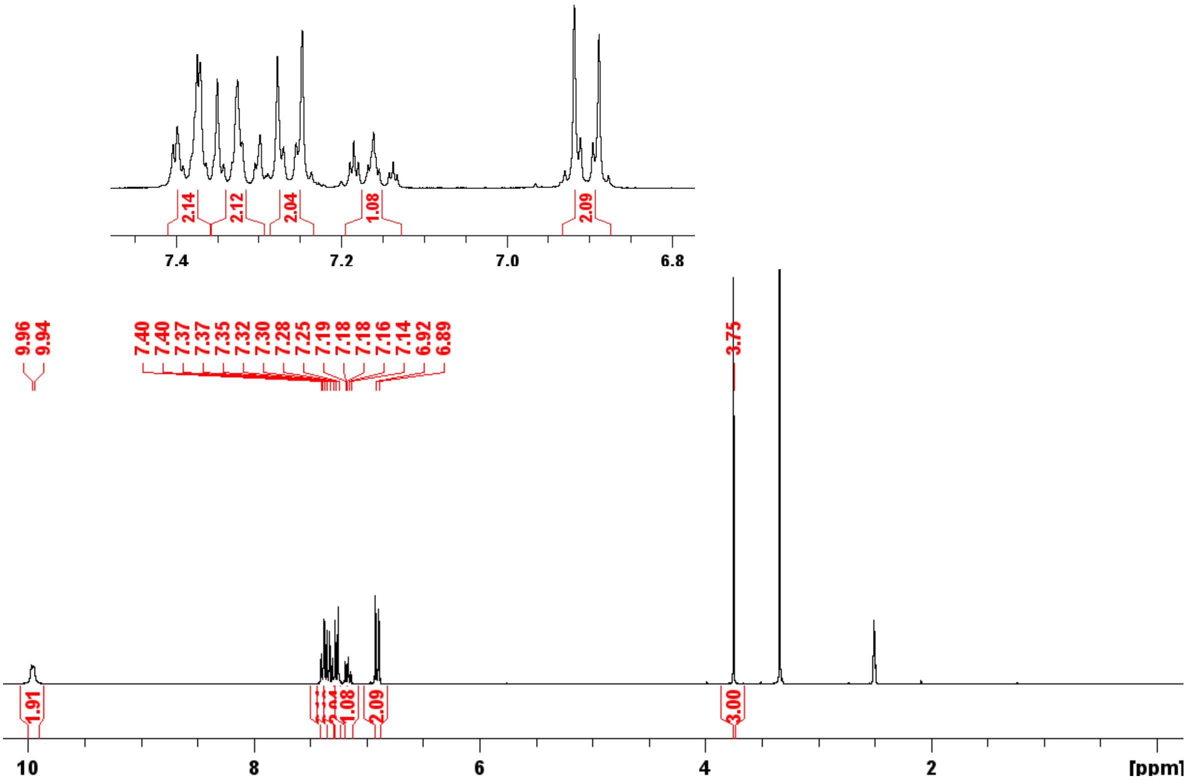

Figure S4. ^{1}H-NMR spectrum of compound **27** (500 MHz, DMSO-d_6)

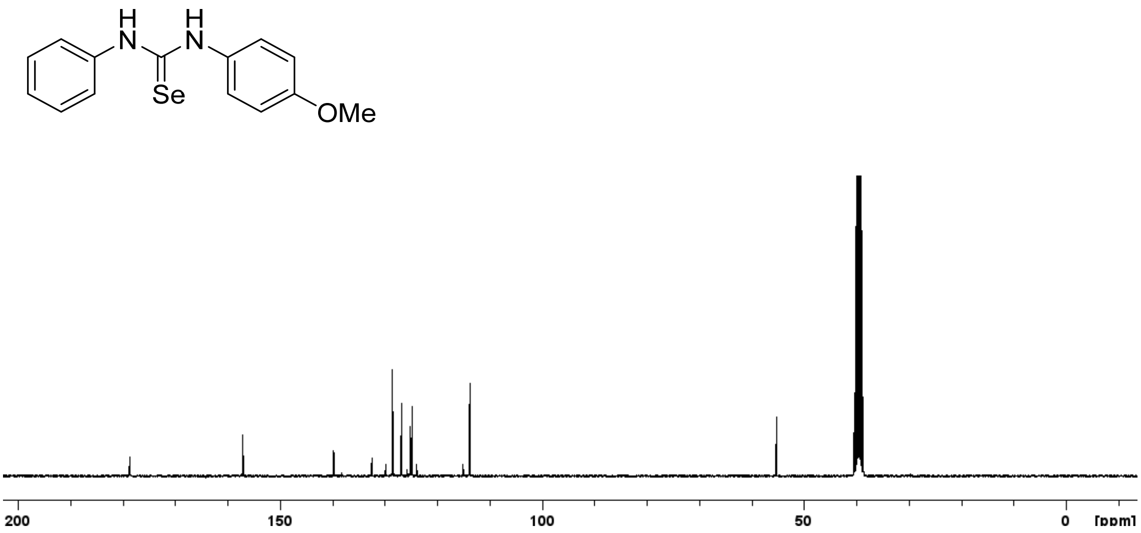

Figure S5. ^{13}C-NMR spectrum of compound **27** (125.7 MHz, DMSO-d_6)

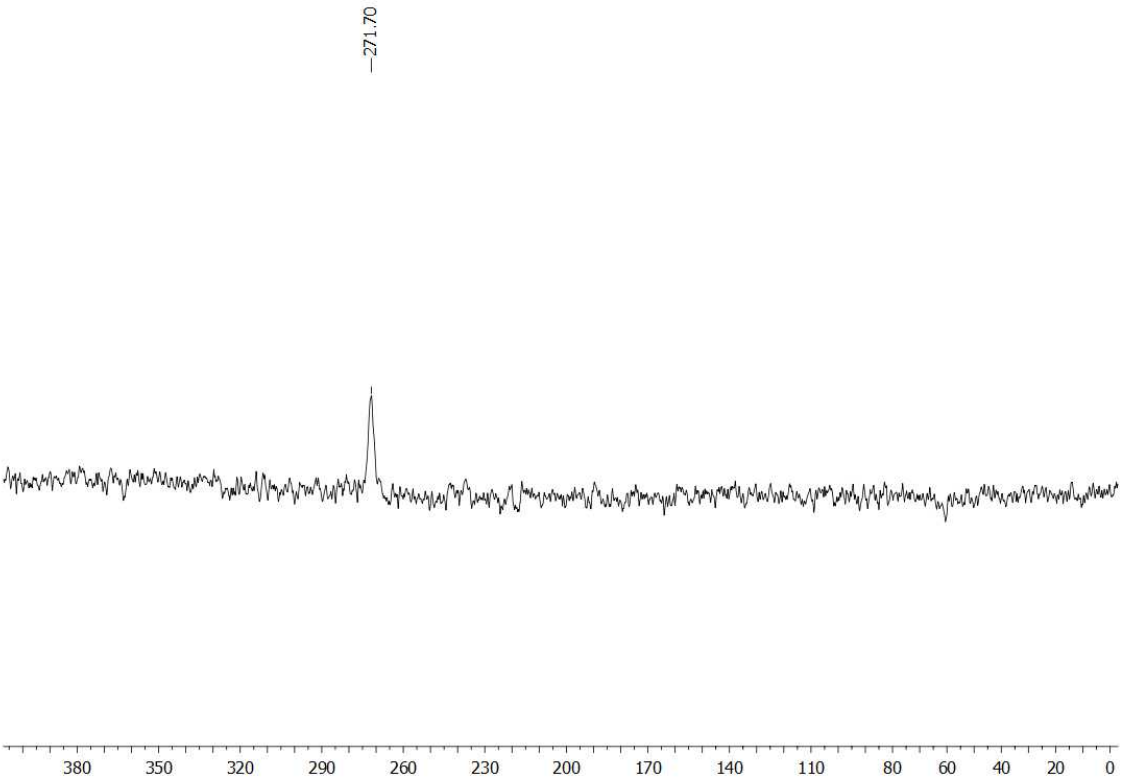

Figure S6. ^{77}Se-NMR spectrum of compound **27** (95 MHz, DMSO-d_6)

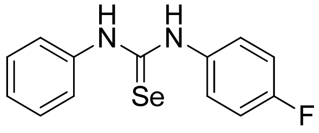

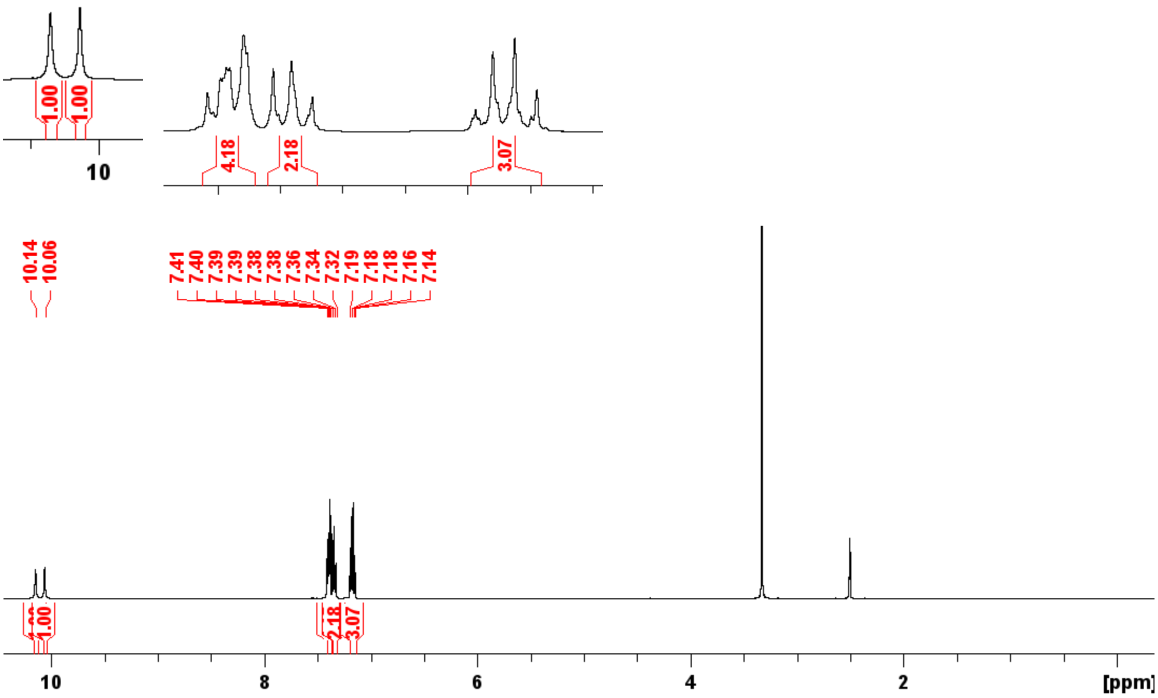

Figure S7. ^{1}H-NMR spectrum of compound **28** (500 MHz, DMSO-d_6)

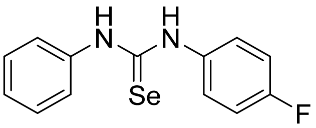

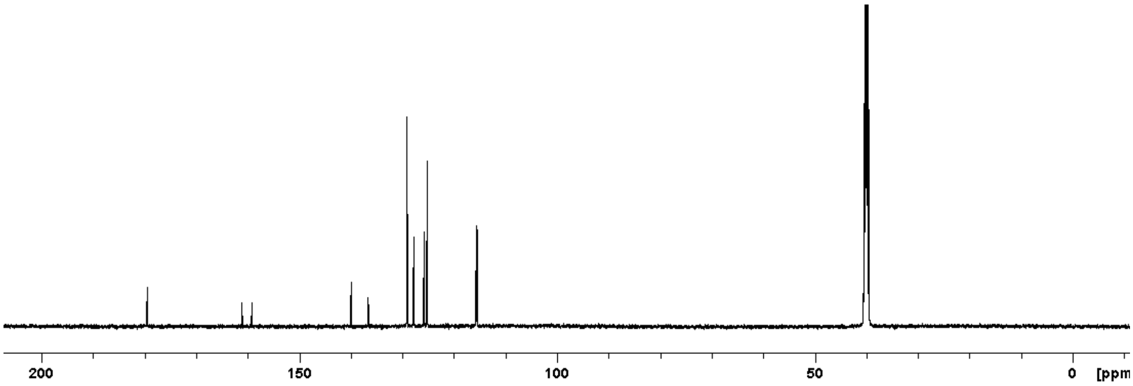

Figure S8. ^{13}C-NMR spectrum of compound **28** (125.7 MHz, DMSO-d_6)

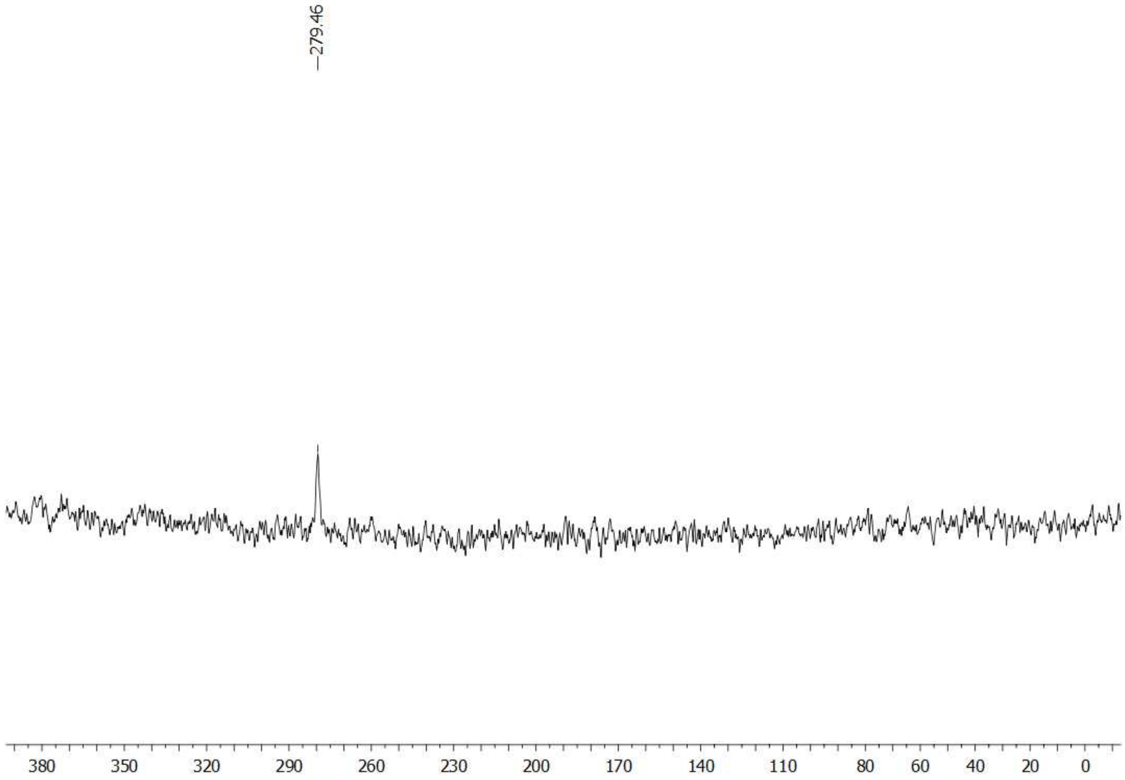

Figure S9. ^{77}Se-NMR spectrum of compound **28** (95 MHz, DMSO-d_6)

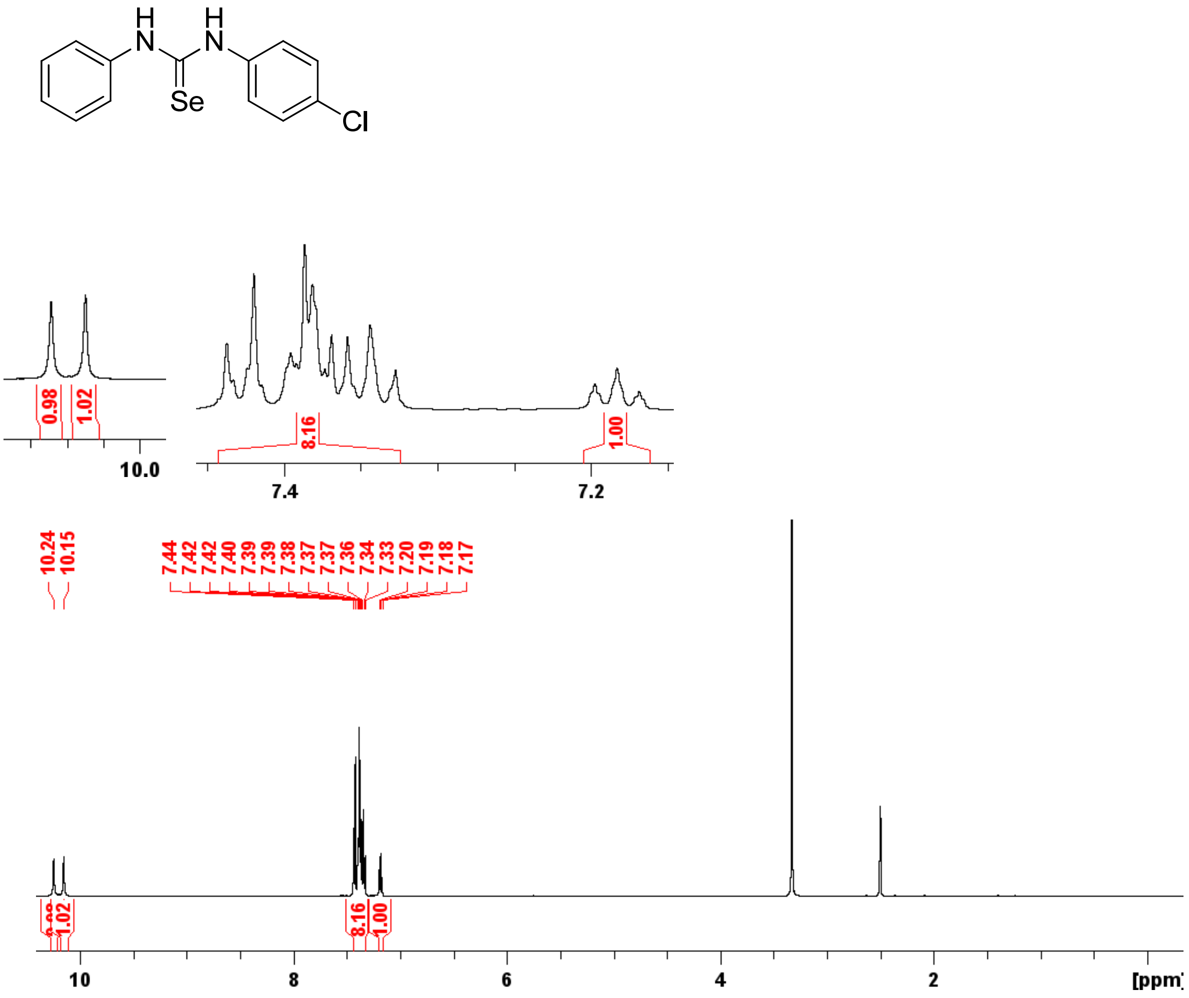

Figure S10. ^{1}H-NMR spectrum of compound **29** (500 MHz, DMSO-d_6)

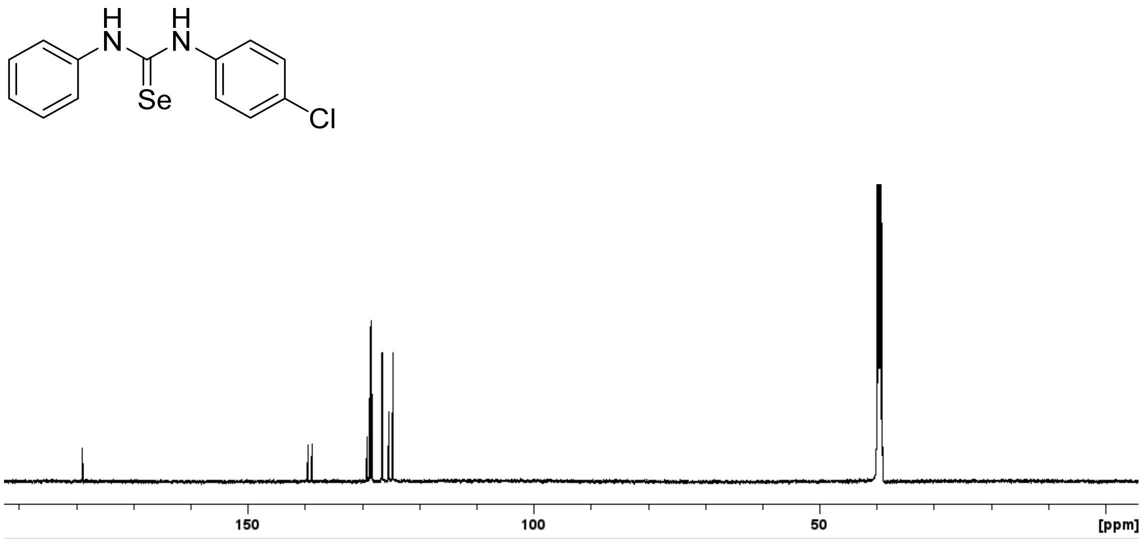

Figure S11. ^{13}C-NMR spectrum of compound **29** (125.7 MHz, DMSO-d_6)

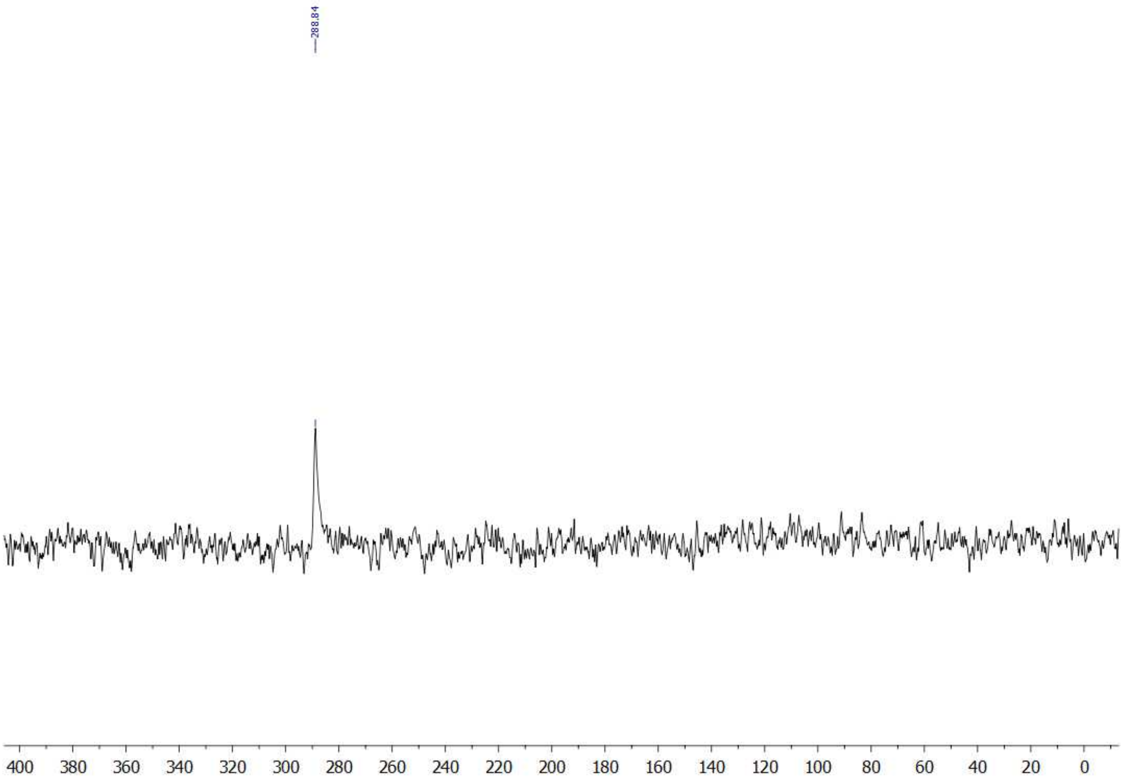

Figure S12. ^{77}Se-NMR spectrum of compound **29** (95 MHz, DMSO-d_6)

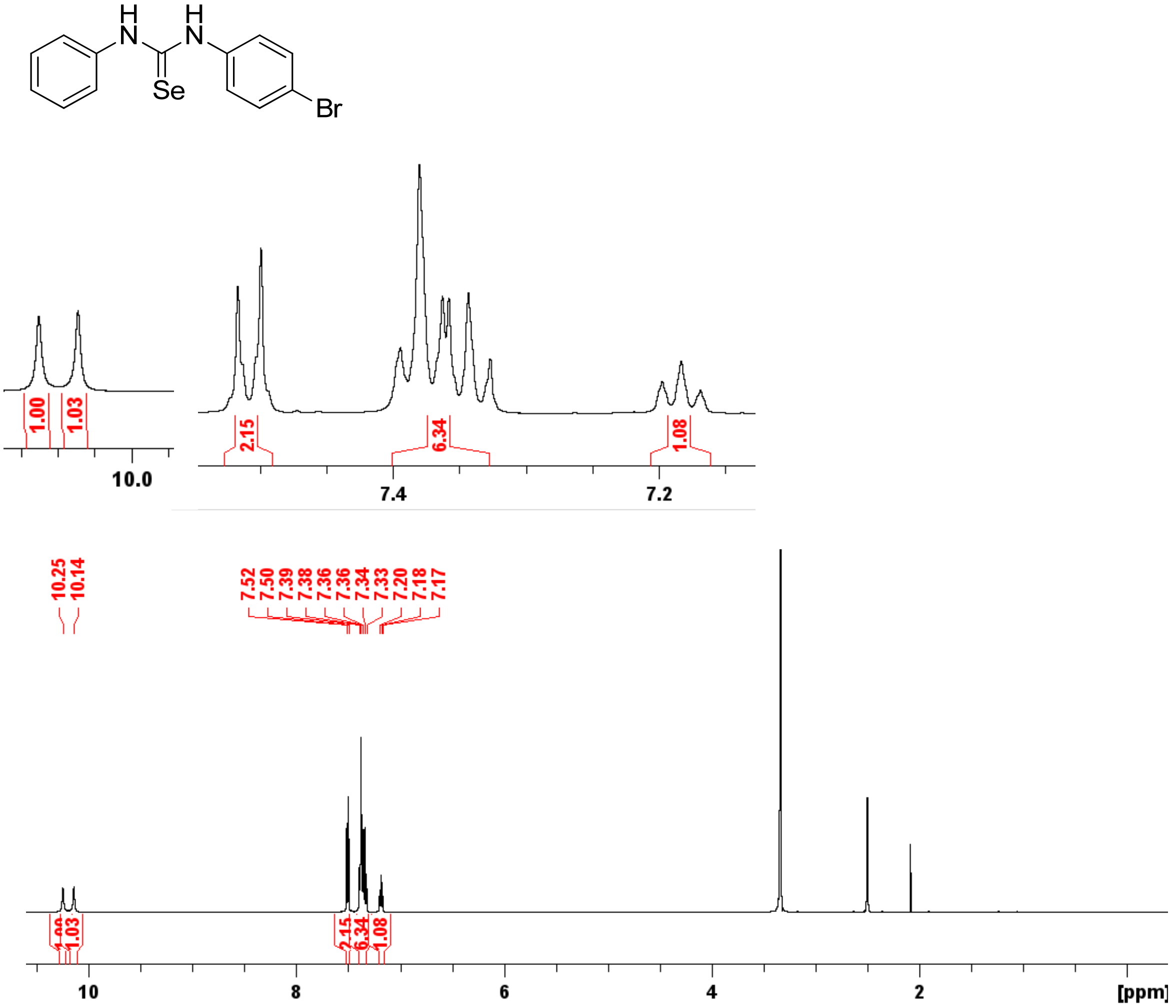

Figure S13. ^{1}H-NMR spectrum of compound **30** (500 MHz, DMSO-d_6)

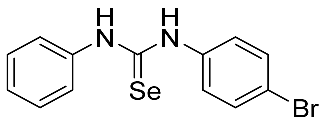

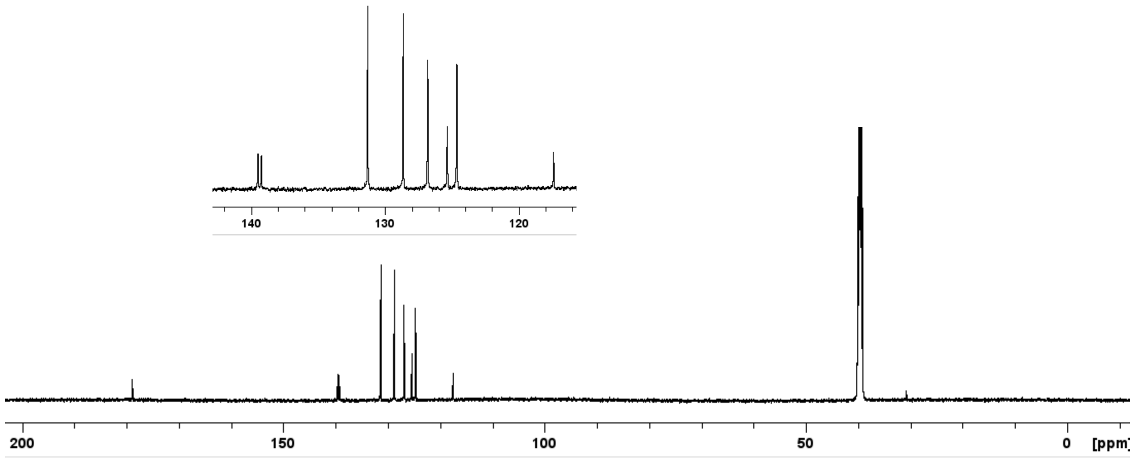

Figure S14. ^{13}C-NMR spectrum of compound **30** (125.7 MHz, DMSO-d_6)

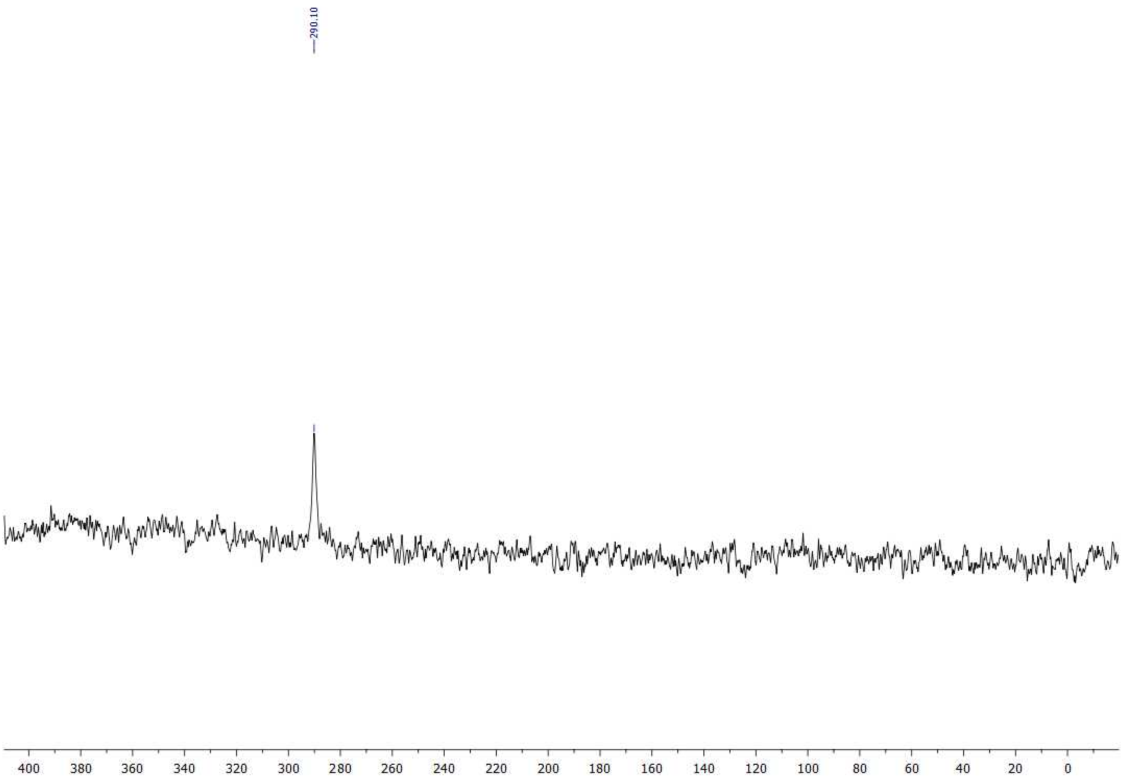

Figure S15. ^{77}Se-NMR spectrum of compound **30** (95 MHz, DMSO-d_6)

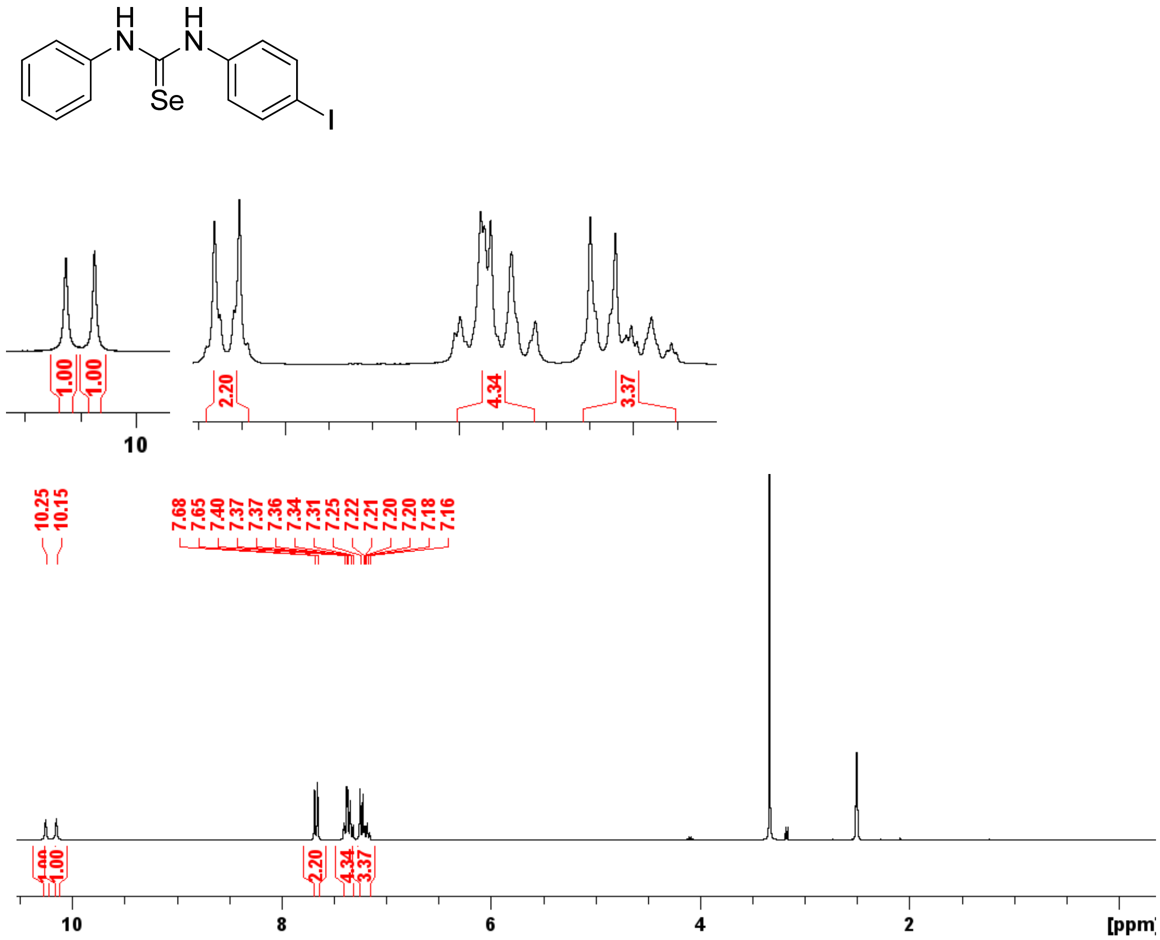

Figure S16. ^{1}H-NMR spectrum of compound **31** (500 MHz, DMSO-d_6)

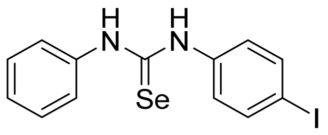

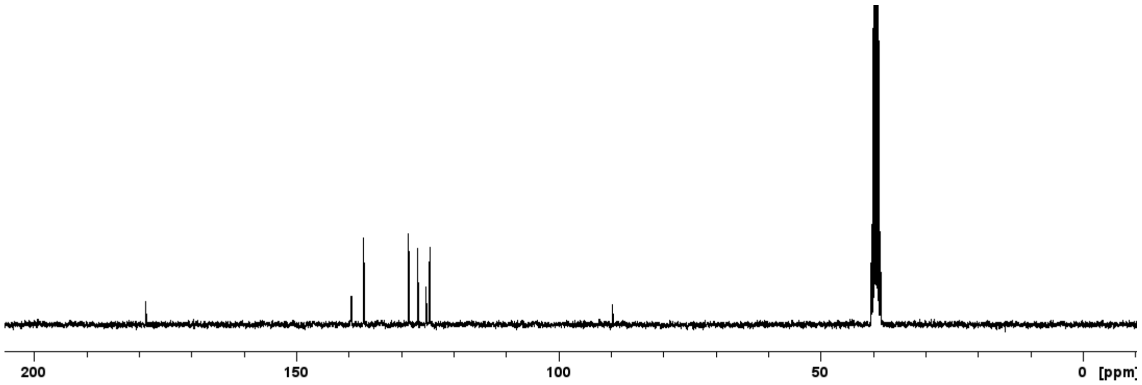

Figure S17. ^{13}C-NMR spectrum of compound **31** (125.7 MHz, DMSO-d_6)

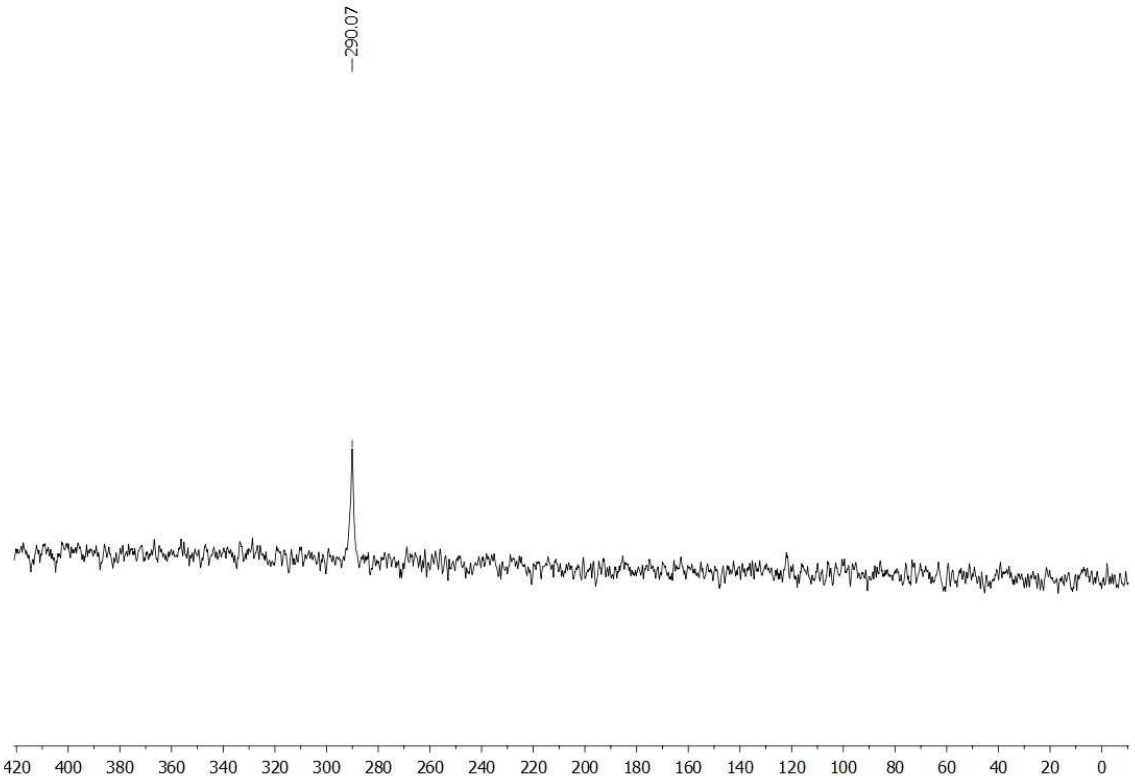

Figure S18. ^{77}Se-NMR spectrum of compound **31** (95 MHz, DMSO-d_6)

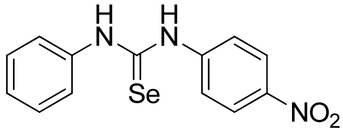

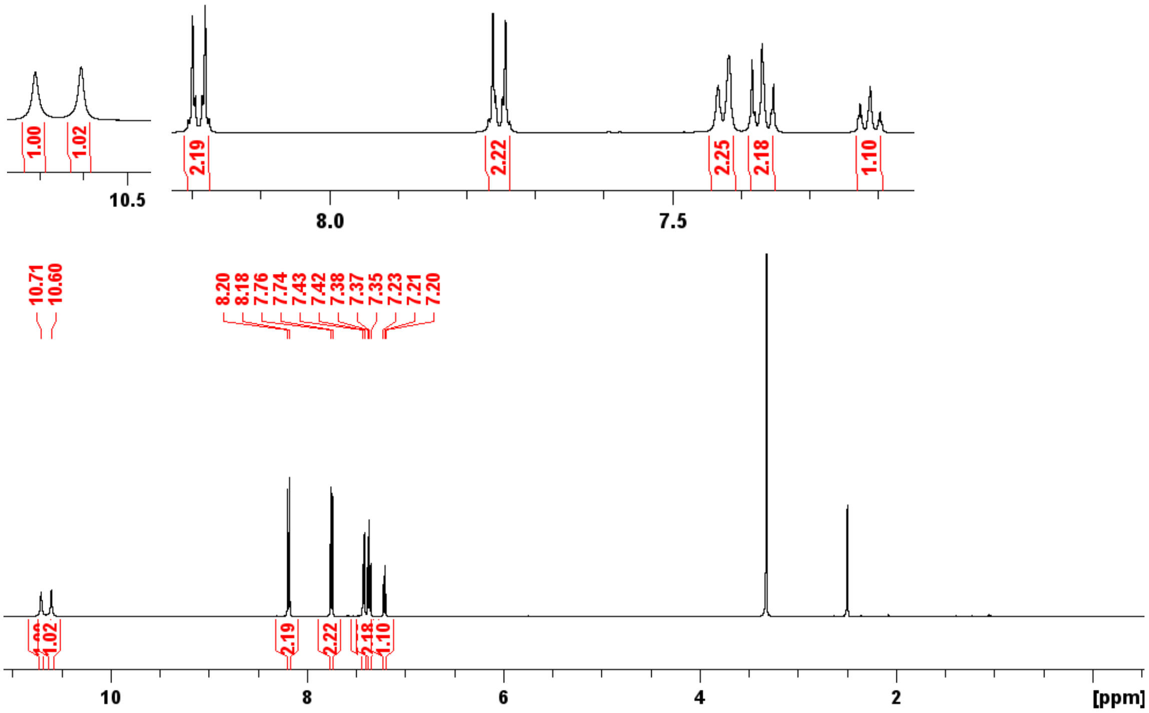

Figure S19. ^{1}H-NMR spectrum of compound **32** (500 MHz, DMSO-d_6)

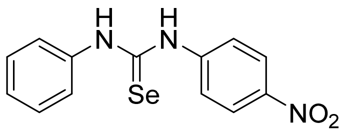

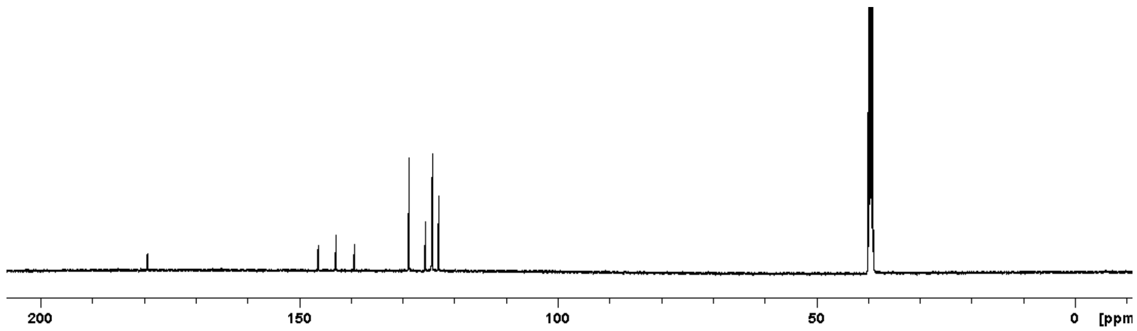

Figure S20. ^{13}C-NMR spectrum of compound **32** (125.7 MHz, DMSO-d_6)

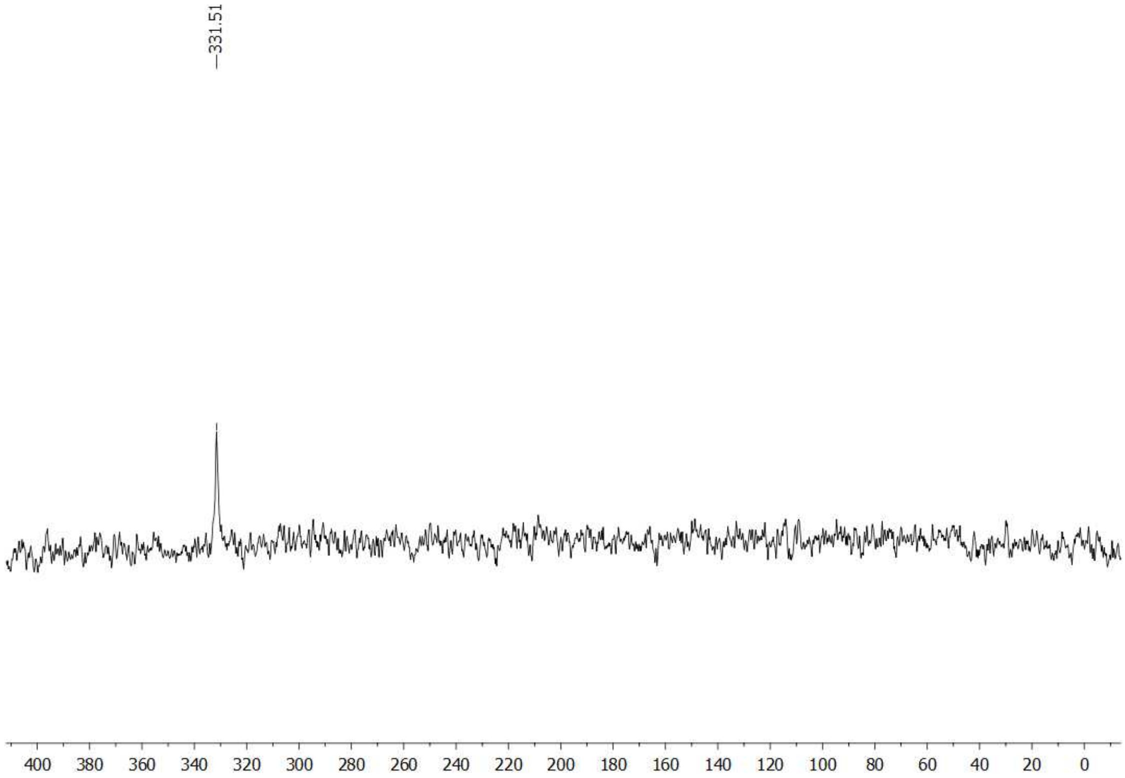

Figure S21. ^{77}Se-NMR spectrum of compound **32** (95 MHz, DMSO-d_6)

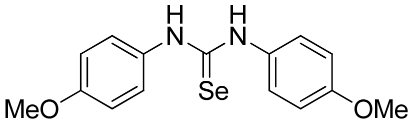

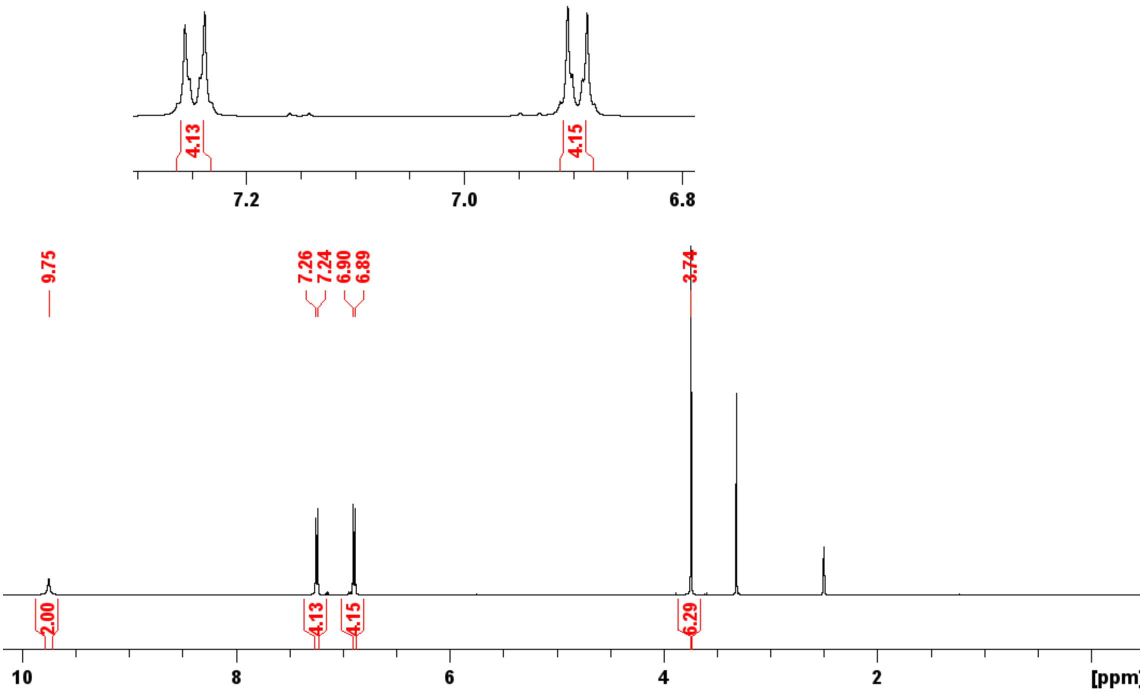

Figure S22. ^{1}H-NMR spectrum of compound **33** (500 MHz, DMSO-d_6)

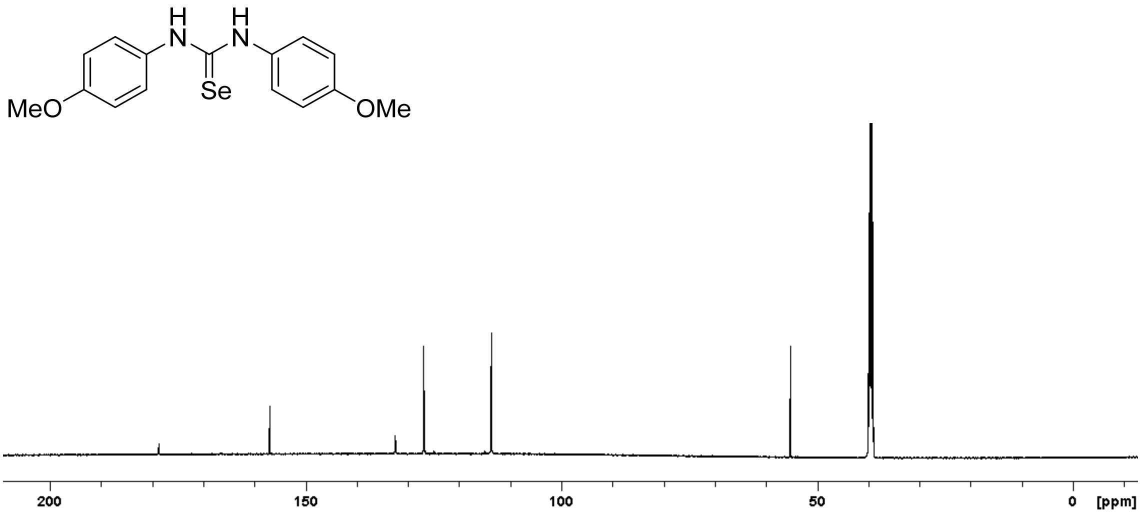

Figure S23. ^{13}C-NMR spectrum of compound **33** (125.7 MHz, DMSO-d_6)

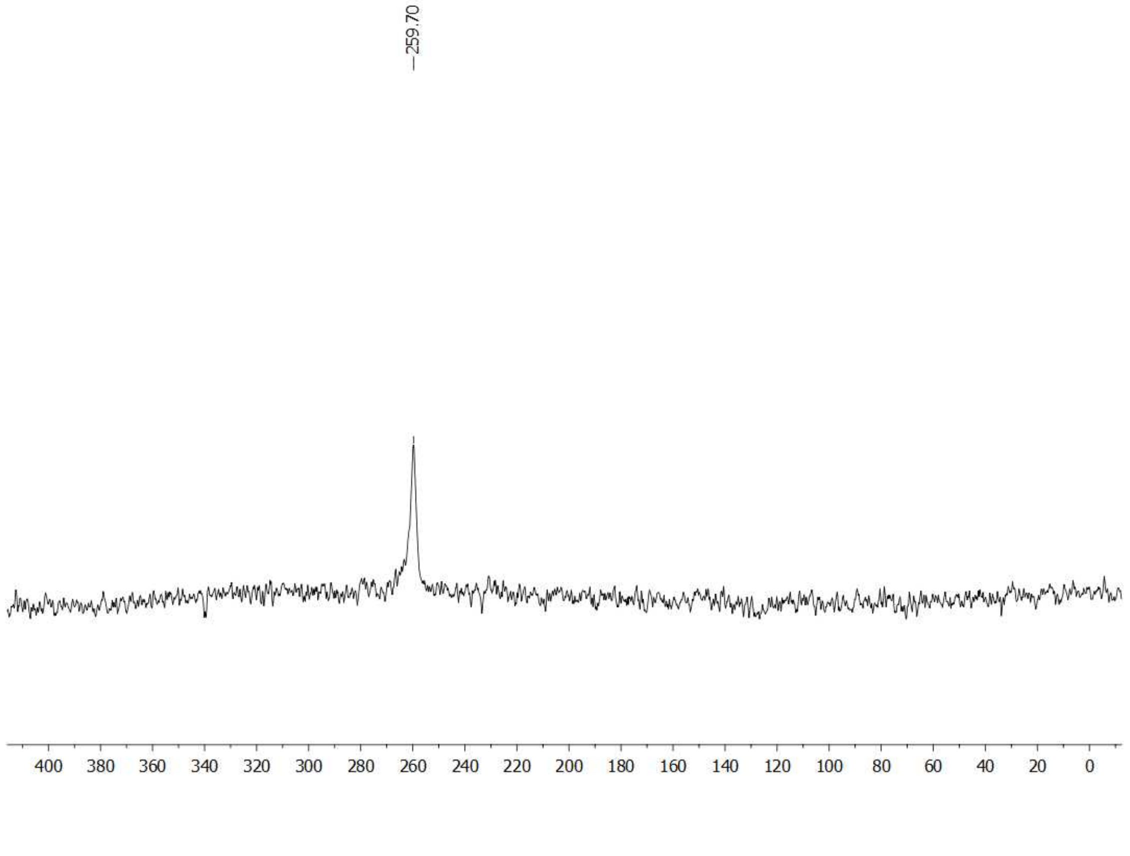

Figure S24. ^{77}Se-NMR spectrum of compound **33** (95 MHz, DMSO-d_6)
